# Anti-Inflammatory Effects of Pingyin Rose Essential Oil in LPS-Induced HaCaT Cells: An in Vitro and in Silico Study

**DOI:** 10.3390/ijms27073174

**Published:** 2026-03-31

**Authors:** Jingyi Song, Rifat Nowshin Raka, Zhongwei Zhang, Junsong Xiao, Mingquan Huang, Hua Wu

**Affiliations:** 1 Beijing Key Laboratory of Plant Resource Research and Development, Beijing Technology and Business University, Beijing 100048, China; 2050301006@st.btbu.edu.cn (J.S.);; 2 Key Laboratory of Brewing Molecular Engineering of China Light Industry, Beijing Technology and Business University, Beijing 100048, China

**Keywords:** skin inflammation, oxidative stress, TLR4-NF-κB pathway, network pharmacology

## Abstract

Pingyin rose essential oil (PREO) is extracted from fresh petals exclusively cultivated in Shandong Province. This PREO has been used in traditional Chinese medicine (TCM) for decades to treat skin issues like excessive oxidative stress and inflammation. The purpose of this study was to assess the impact of PREO on the inflammatory pathway in HaCaT cells produced by LPS. In vitro methods were used to ascertain the expression of inflammatory proteins, and network pharmacological analysis was employed to predict the signaling pathway. According to our findings, PREO significantly reduced LPS-induced oxidative stress, decreasing nitric oxide (NO) and reactive oxygen species (ROS) production by 42% and 38%, respectively, and malondialdehyde (MDA) levels by 35%, while enhancing superoxide dismutase (SOD) activity by 28% (*p* < 0.01). PREO treatment (0.1%, 18 h) markedly suppressed pro-inflammatory cytokines, with mRNA levels of TNF-α, IL-1β, IL-6, and IL-8 reduced by 52%, 47%, 45%, and 40%, respectively. Mechanistically, PREO inhibited the TLR4-NF-κB pathway, downregulating MyD88 and TRIF expression by 60% and 55%, and reducing NF-κB p65 and IκB-α phosphorylation by 50% and 48%. Network pharmacology and molecular docking identified Citronellol (54.37% of PREO) as the major bioactive component, exhibiting strong binding affinities with IKKβ (−5.7 kcal/moL) and MyD88 (−4.5 kcal/moL). This research, distinct from previous investigations on Rosa rugosa polyphenols, provides a novel mechanistic link between PREO’s traditional use and its observed anti-inflammatory and antioxidant effects in keratinocytes, specifically through inhibition of the TLR4-NF-κB pathway.

## 1. Introduction

Epidermal inflammation is the primary cause of skin health impairment. As the interface between the body and the external environment, the skin must deal with external harmful stimuli, including pathogenic microorganism invasion, stress, radiation, etc. [1]. Generally, there is a balance between pro- and anti-inflammatory cytokines in a healthy body, but the overproduction of pro-inflammatory mediators will break the equilibrium and cause oxidative reactions [2]. This phenomenon causes skin lesions and eventually leads to epidermal inflammation. Overproduction of inflammatory mediators such as tumor necrosis factor-alpha (TNF-α), interleukins (IL-1β, IL-6, IL-10), nitric oxide (NO), and reactive oxygen (ROS) secreted by local cells plays a key role in this imbalance [3]. This is the reason for follicular epithelium damage and melanin deposition induction [4]. However, the underlying signaling pathway of skin inflammation is complex and needs to be explored. Current anti-inflammatory therapies, including NSAIDs and antibiotics, are limited by significant drawbacks. Long-term NSAID use correlates with gastrointestinal complications in 15–30% of patients and cardiovascular risks [5,6], while antibiotic overuse has driven antimicrobial resistance (AMR), with *Staphylococcus aureus* resistance rates exceeding 40% in some regions [7,8]. These limitations underscore the urgency for safer, multi-target alternatives.

Traditional Chinese Medicine (TCM) boasts a rich history of anti-inflammatory medication that includes a variety of natural compounds. As skin inflammation is susceptible to frequent exposure to harmful stimulators, suppressing inflammatory pathways and neutralizing oxidation are promising approaches to treat inflammation [9,10,11,12]. Pingyin rose, especially its flower bud, is a well-represented component of TCM for its fragrance and therapeutic efficacy. It has been natively cultivated in Pingyin town for 1300 years, and in 2021, it was chosen as the “second city flower of Jinan”. There is different scientific evidence proving different bioactivities of *Rosa rugosa* [13,14,15]. Pingyin Rose Essential oil (PREO), derived from the fresh flowers of Pingyin rose, has an enriched phytochemical profile and is characterized by a high Citronellol content of around 54%, a monoterpenoid with well-documented anti-inflammatory and antioxidant properties [16,17]. Unlike oils from Lavandula or Melaleuca, which often require higher concentrations for efficacy and carry risks of dermal irritation, PREO exhibits a favorable safety profile in cosmetic applications. Furthermore, its historical use in TCM for over 1300 years provides empirical validation of its therapeutic potential, while modern studies confirm its dual modulation of oxidative and inflammatory pathways in preclinical models [18,19].

Network pharmacology is a combined science of system biology and computational biology to construct a drug-disease network, to find potential targets as well as active ingredients, and to suggest potential drug components by computer-aided drug designing [19]. Molecular docking is a part of this multidisciplinary field as it defines the forces and interaction base between drug and target [20]. Compared with our previous macrophage-based investigations, the present work specifically focuses on keratinocyte-mediated cutaneous inflammation, thereby providing epidermis-relevant mechanistic evidence for PREO. In this study, we analyzed the chemical composition of PREO, identified active components and probable targets, and performed molecular docking with the significant compounds as ligands for the core target. Lipopolysaccharide (LPS) was employed to induce the keratinocyte cell line HaCaT to assess the anti-inflammatory efficacy of PREO against skin inflammation. The results showed that PREO can reduce LPS-stimulated cell inflammation by reducing pro-inflammatory cytokine expression. We additionally looked into the possibility that PREO’s ability to inhibit oxidative stress and the TLR4-NF-κB pathway is linked to its anti-inflammatory properties.

## 2. Results

### 2.1. Effects of LPS and PREO Individually on HaCaT Cells

**Figure 1:**
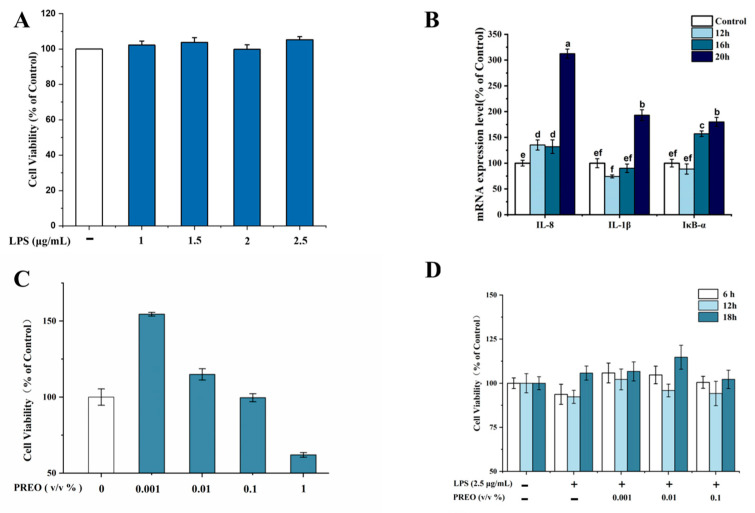
Effects of PREO/LPS on HaCaT cells (**A**) Effects of LPS on HaCaT cell viability. (**B**) The effects of LPS on mRNA levels in HaCaT cells. (**C**) The effects of PREO on HaCaT cell viability. (**D**) The effects of PREO on cell viability in LPS-induced HaCaT cells. Values represent means ± SD, *n* = 3. *p* < 0.01. “a–f” signifies the value by the Waller–Duncan test.

To establish a reliable inflammatory cell model, we first evaluated the effects of LPS and PREO treatment at varying concentrations on HaCaT cell viability and oxidative stress using the MTT assay. As shown in Figure 1A, LPS up to 2.5 μg/mL does not have any cytotoxicity. The expression of pro-inflammatory cytokines in different incubation periods determined the oxidative stress level. After 20 h of incubation, IL-8, IL-1β, and IκB-α mRNA expressions were almost doubled compared to the control (no-LPS) (Figure 1B). However, LPS can induce oxidative stress in HaCaT cells in a dose-time-dependent manner. In the case of PREO-induced MTT assay, cells show a decrease in viability with the increase in concentration after 24 h. According to Figure 1C, the highest concentration was 1%, which is visibly toxic to cells. Therefore, LPS 2.5 μg/mL and 0.001–0.1% (*v*/*v*) PREO had been used for further explorations.

### 2.2. Effects of PREO on LPS-Induced HaCaT Cell Viability

Following validation of the inflammatory model, we screened PREO’s effective concentration and treatment duration, then assessed its impact on cell viability using the MTT assay. After being stimulated with 2.5 μg/mL of LPS for 20 h, the cells were treated with 0.001–0.1% (*v*/*v*) PREO for 6–18 h to determine the protective effects of PREO on LPS-induced cell viability loss. LPS showed a slight decrease in cell viability after 6 h of induction, which was regained after 18 h, probably because of cell adaptation. Post-LPS induction (2.5 μg/mL, 20 h), cells treated with 0.001–0.1% PREO for 6–18 h showed restored viability (>90%), suggesting adaptation and protective efficacy (Figure 1D).

### 2.3. Effects of PREO on NO and ROS Production in LPS-Induced HaCaT Cells

**Figure 2:**
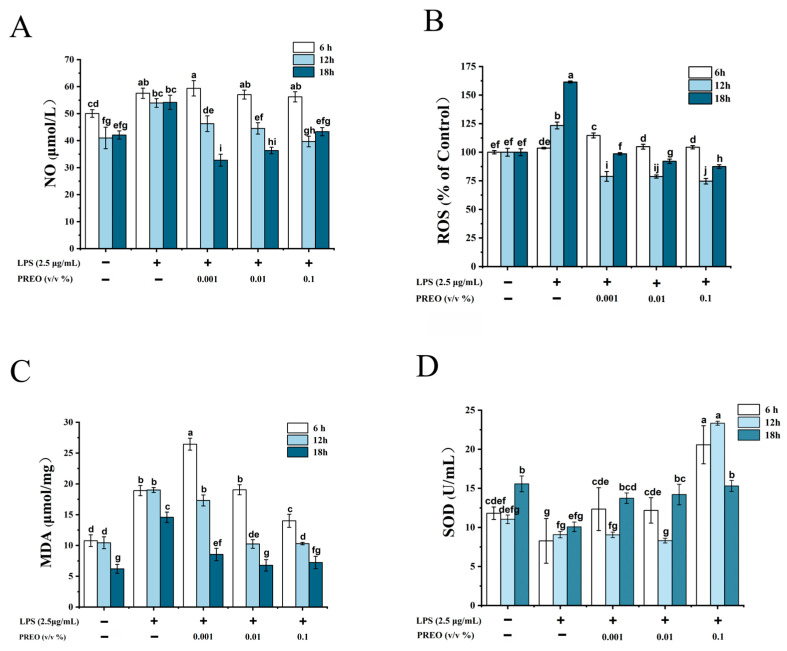
The effects of PREO on oxidative stress in LPS-induced HaCaT cells. (**A**,**B**) The effects of PREO on intracellular NO and ROS production in LPS-induced HaCaT cells. (**C**,**D**) The effects of PREO on MDA production and SOD activity in LPS-induced HaCaT cells. Values represent means ± SD, *n* = 3. *p* < 0.01. “a–j” signifies the value by the Waller–Duncan test.

Increased NO and ROS are the primary indicators of oxidative stress in our body. PREO showed a positive effect on both markers. As shown in Figure 2A, the levels of NO markedly increased in response to 20 h of induction of LPS. Treatment with 0.001%, 0.01%, and 0.1% PREO showed no effects on the NO level after 6 h, but after 12 h of induction, PREO reduced the NO level notably, and after 18 h, 0.001% and 0.1% PREO decreased it further. PREO treatment (0.1%, 18 h) significantly reduced LPS-induced NO production by 42% compared to LPS-treated cells (*p* < 0.01, Figure 2A).

LPS also increased the ROS level to a high range in the cell. Conversely, treatment with 0.001%, 0.01%, and 0.1% PREO for 12 h significantly reduced ROS levels, but both 6 h and 18 h incubation showed a slight rise compared to the control (Figure 2B). ROS levels in LPS-stimulated HaCaT cells decreased by 38% following 0.1% PREO treatment for 18 h (*p* < 0.01, Figure 2B). These results indicate that PREO can possibly restore the endogenous antioxidant defense mechanisms impaired by LPS. The effects were related to the concentration of PREO and induction time.

### 2.4. Effects of PREO on Activity of SOD and Production of MDA

SOD and MDA are the key markers of oxidative stress. Respective kits measured the levels of SOD activity and MDA content. In LPS-induced HaCaT cells, SOD activity was lowered as shown in Figure 2C,D, but it was noticeably elevated (28%) at 0.1% PREO treatment for 6 h, and it then decreased after 18 h (relative to 6 h) of exposure. After 20 h of LPS stimulation, the MDA levels markedly rose, while PREO therapy significantly decreased them (35%) in a time- and dose-dependent way. It is possible that the treatment period and concentration were too short for PREO administered at 0.001% concentration for 6 h to have any effect on MDA production. These findings demonstrated that PREO could reduce oxidative stress and thereby modify the impact of inflammation.

### 2.5. Effects of PREO on Inflammatory Cytokines Production in mRNA Level

**Figure 3:**
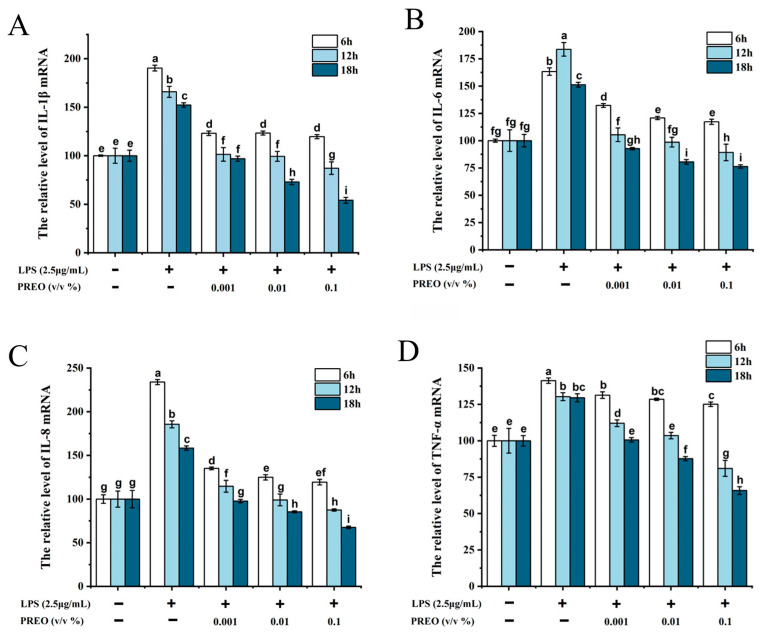
The effects of PREO on pro-inflammatory cytokines expression in LPS-induced HaCaT cells. (**A**) IL-1β. (**B**) IL-6. (**C**) IL-8. (**D**) TNF-α. Values represent means ± SD, *n* = 3; *p* < 0.01. “a–i” signifies the value by the Waller–Duncan test.

One of the most demonstrable initial changes due to inflammation is the increase in inflammatory cytokine expression levels. IL-1β, IL-6, IL-8, and TNF-α are the most common cytokines overexpressed due to inflammation. As shown in Figure 3, the mRNA production of IL-1β, IL-6, IL-8, and TNF-α in LPS-induced HaCaT cells markedly increased in response to the LPS (*p* < 0.05) compared to the control group, both after 6 h and 18 h of induction. Treatment with 0.001%, 0.01%, and 0.1% PREO significantly reduced LPS-induced mRNA levels of IL-1β (47% reduction, *p* < 0.01, Figure 3A), IL-6 (45% reduction, *p* < 0.01, Figure 3B), IL-8 (40% reduction, *p* < 0.01, Figure 3C), and TNF-α (52% reduction, *p* < 0.01, Figure 3D) in a time- and dose-dependent manner compared to LPS-exposed HaCaT cells. After 6 h of treatment, PREO significantly decreased the transcription level of the cytokines, while after 12 h and 18 h of inductions caused more reductions with time. A total of 0.001% PREO showed the same results as the control after 18 h of incubation, following further decrease with 0.01% and 0.1% PREO. Together, the above results indicate that PREO is a potent inhibitor of LPS-induced inflammatory cytokine expression.

### 2.6. Effects of PREO on TLR4-NF-κB Pathway in LPS-Induced HaCaT Cells

#### 2.6.1. TLR4-NF-κB Pathway-Related Gene Expression

**Figure 4:**
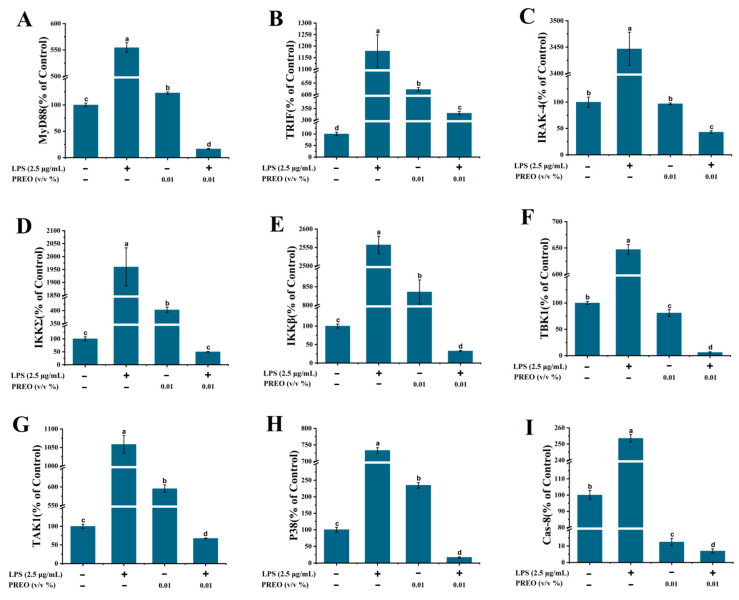
The effects of 0.01% PREO on mRNA levels of TRL4 pathway Proteins in HaCaT cells with or without LPS. (**A**–**I**) Effects of PREO on the MyD88, TRIF, IRAK-4, IKKΣ, IKKβ, TBK1, TAK1, P38 and Casp-8 mRNA expression levels, respectively. Values represent means ± standard error of the mean, *n* = 3; *p* < 0.01. “a–d” signifies the value by the Waller–Duncan test.

As LPS is a well-known ligand of TLR4, we evaluated the level of mRNA expression involved in this pathway. Our network pharmacological analysis indicated the direct involvement of the TLR4-NF-κB pathway. Therefore, for further investigation, MyD88, TBK1, Caspase-8, TRAK-4, TAK1, IKKβ, IKKΣ, P38, and TRIF mRNA expression levels were measured using RT-PCR analysis. The chosen treatment for the cell were 0.01% PREO for 18 h following 20 h of LPS pre-induction. The cell was also exposed to only 0.01% PREO to check its individual effects (Figure 4). LPS significantly raises the expression level for all mRNA compared to the control. This is because LPS increases its expression. However, 18 h of exposure with 0.01% of PREO on LPS-induced cells relatively lowers the inflammation level. When cells were treated with 0.01% PREO without LPS, TAK1, IKKβ, IKKΣ, P38, and TRIF mRNA expression levels increased compared to normal cells. MyD88, TBK1, and TRAK-4 expression had slight changes, while Casp8 showed decreased expression in LPS-free PREO-induced cells.

#### 2.6.2. TLR4-NF-κB Pathway-Related Proteins Expression

**Figure 5:**
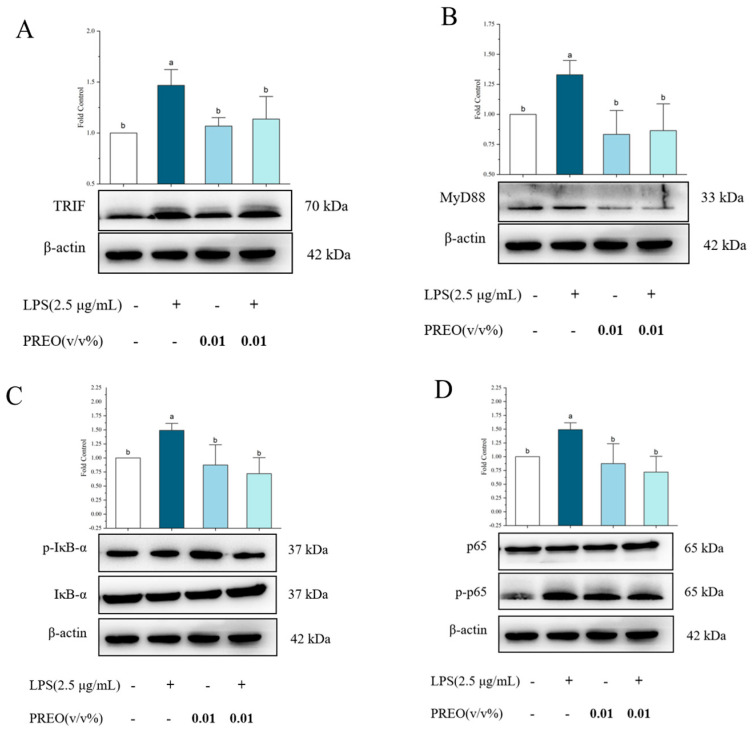
The effects of PREO on inflammatory protein levels at 18 h in LPS-induced HaCaT cells. (**A**) TRIF protein expression. (**B**) MyD88 protein expression. (**C**) IκB-alpha protein expression. (**D**) NF-κb p65 expression. Values represent means ± standard error of the mean; *p* < 0.01. “a–b” signifies the value by the Waller–Duncan test. All original bands are provided as Supplementary Data.

The production of inflammatory mediators is strongly affected by TLR4-NF-κB pathways in the HaCaT cells. MyD88, TRIF, p65, and IκB-α are the major components of NF-κB activated by LPS in the HaCaT cells. As shown in Figure 5, LPS significantly upregulated the expression of MyD88 and TRIF proteins in HaCaT cells. In contrast, treatment with 0.01% PREO effectively inhibited the expression of both proteins (Figure 5A,B). Specifically, LPS remarkably increased the protein expression of p65 and IκB-α and promoted their phosphorylation. Furthermore, 0.01% PREO significantly reduced the expression of p65 and IκB-α and inhibited the phosphorylation of both proteins in HaCaT cells after 18 h of incubation (Figure 5C,D). Additionally, LPS significantly decreased the phosphorylation of P38 MAPK protein (Figure 5C). These experimental findings suggest that PREO is likely to inhibit the TLR4 signaling pathway by suppressing the production of MyD88 and TRIF protein both, thereby controlling the activation of the downstream NF-κB pathway. Furthermore, PREO can mediate the inflammation induced by LPS in HaCaT cells via the NF-κB pathway.

### 2.7. Network Pharmacology

To unravel the mechanistic basis of PREO’s anti-inflammatory effects, we integrated network pharmacology and molecular docking to identify key targets within the TLR4-NF-κB pathway. Citronellol is the main part of PREO, making up 54.37% of it. We found that Citronellol binds well to two proteins, IKKβ and MyD88. The binding energies were −5.7 kcal/moL for IKKβ and −4.5 kcal/moL for MyD88. These binding results suggest that Citronellol might regulate NF-κB activation.

#### 2.7.1. Main Active Components and Druggable Targets of PREO

**Figure 6:**
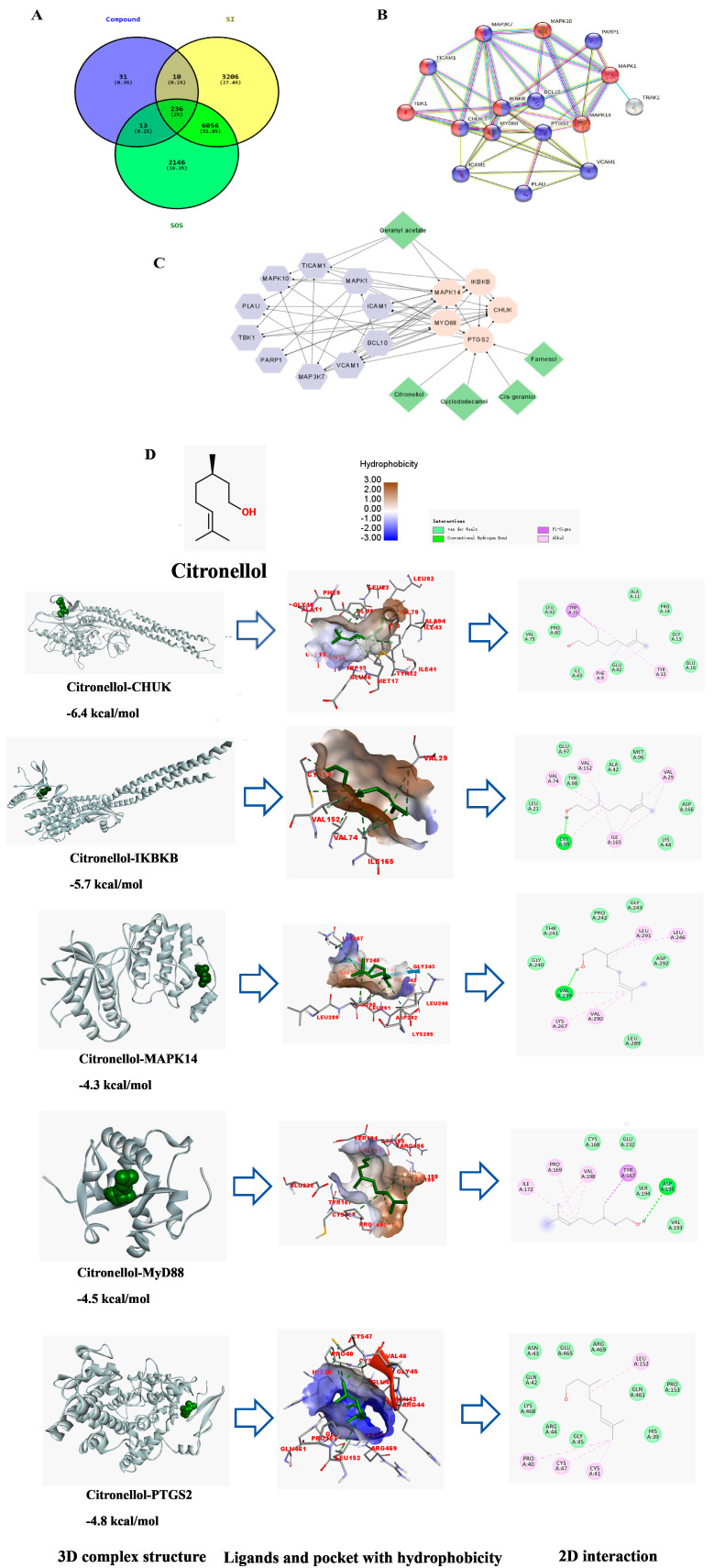
Network pharmacology studies by constructing a “compound-target” pathway and Molecular docking interaction of Citronellol with target proteins in 3D and 2D models. (**A**) Ven-diagram of targets. (**B**) PPI network. (**C**) Highest degree-based compound-target network. (**D**) Citronellol-Protein interaction based on molecular docking.

Using criteria of >25% OB, 24 components were selected initially. Depending on their potential targets, 5 active components were determined (Table 1). A total of 236 common targets were identified between CT, SI, and SOS. After sorting out the targets using NCBI and UniProtKB, 29 targets were chosen upon intersection. A big network was first constructed with 54 nodes and 157 edges. Using a cystoscope, the top five targets were filtered using a degree value > 10. As shown in Figure 6, 20 nodes and 58 edges were finally found after merging the final targets with top degree scores and edge betweenness. IKBKB was determined as the top and centered target following CHUK, MyD88, MAPK14, and PTGS2.

#### 2.7.2. Molecular Docking Analysis

All 5 proteins were energy minimized and prepared for docking. Citronellol (compound ID = 8842) was used as ligands because of its high percentage in PREO, along with a high ranking from network pharmacological analysis. The binding affinities with Citronellol were: CHUK −6.4 kcal/moL, IKBKB −5.7 kcal/moL, MAPK14 −4.3 kcal/moL, MyD88 −4.5 kcal/moL, and PTGS2 −4.8 kcal/moL. All the docking results were presented in Table 2. The interactions were presented in 2D format for docked models in Figure 6D.

#### 2.7.3. Analysis of RMSD, RMSF, Rg, SASA, and MM/PBSA

**Figure 7:**
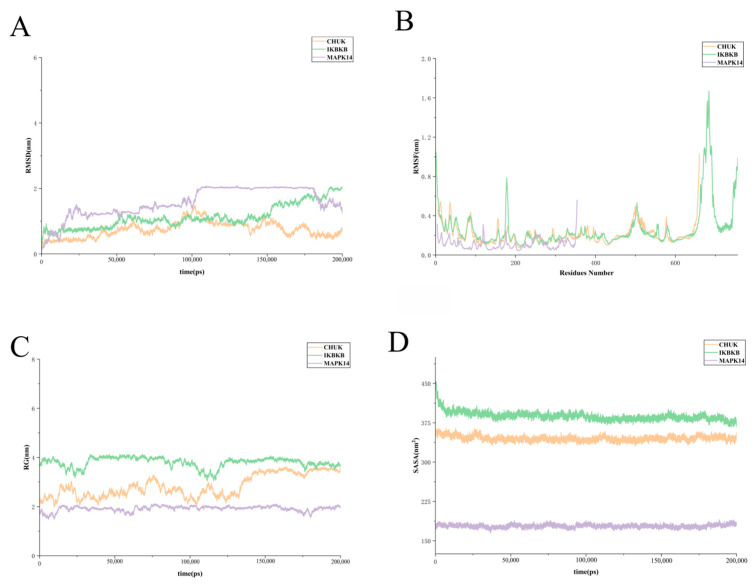
MD simulations of CHUK, IKBKB, and MAPK14 over 200 ns. (**A**) RMSD. (**B**) RMSF. (**C**) Rg. (**D**) SASA.

MD simulations of CHUK, IKBKB, and MAPK14 over 200 ns revealed distinct conformational behaviors. CHUK exhibited the highest structural stability, with minimal RMSD (Figure 7A) deviations, whereas IKBKB and MAPK14 showed notable increases after 100 ns, suggesting late-stage conformational adjustments. RMSF (Figure 7B) analysis indicated substantial flexibility in IKBKB near residue 680 (~1.6 nm), likely reflecting a mobile C-terminal region, while MAPK14 remained locally rigid (<0.3 nm), with deviations primarily arising from rigid-body domain motions. The Rg (Figure 7C) analysis showed clear differences in structural compactness among the three proteins during the 200 ns simulation. MAPK14 remained the most compact and stable, with Rg values around 1.8–2.1 nm. IKBKB had the highest Rg values (3.7–4.1 nm), indicating a larger and less compact structure, although it stayed relatively stable overall. CHUK showed intermediate Rg values but exhibited a gradual increase in the later stage of the simulation, suggesting some conformational expansion. Overall, MAPK14 was the most compact, IKBKB the least compact, and CHUK showed the most noticeable structural change. SASA (Figure 7D) profiles confirmed that all proteins retained their folded conformations, with IKBKB exhibiting higher solvent exposure (~385 nm^2^) and MAPK14 remaining most shielded (~180 nm^2^).

## 3. Discussion

Since the skin is the outermost protective layer of our body, it is the organ most vulnerable to external factors. The epidermis is sensitive to harmful and pathogenic stimuli, triggering mild to severe problems nearly every day. The type and duration of stimulation can have a significant impact on skin damage [21]. The present research focuses on the beneficial impact of PREO on minimizing oxidative stress on the epidermis and improving skin health through the downregulation of inflammatory pathways.

In this study, we used LPS (an endotoxin produced by Gram-negative bacteria) to stimulate HaCaT cells. As a classic pro-inflammatory factor, LPS can regulate the production of intracellular substances through specific signaling pathways, and it is reported to enter the body via toll-like receptor 4 (TLR4) to activate various cells, including keratinocytes such as HaCaT cells [22,23]. Various pro-inflammatory factors (IL-1β, IL-6, IL-8, and TNF-α) and oxidative stress markers are produced as a result of disturbing redox hemostasis and increasing the production of NO and ROS, leading to organ failure [24,25]. Reversing the expression of inflammatory cytokines by downregulating the corresponding pathways is one of the main strategies for reducing inflammation. A more effective remedy for treating inflammation these days is a natural product.

PREO is a proprietary ingredient used in China’s food and cosmetics sectors. Excellent anti-inflammatory and anti-oxidative properties make it a remarkable member of TCM that warrants further investigation [18].

For in vitro validation, we used three different concentrations of PREO treatment for 6, 12, and 18 h against LPS stimulation. A total of 0.1% PREO reduced the inflammation more remarkably than the other two concentrations, even after 18 h of LPS exposure. PREO consistently showed a time-dose-dependent effect on LPS-induced HaCaT cells. It successfully decreased NO, ROS, and MDA levels and increased SOD activity, which consequently lowered the oxidative stress in the cell. Our previous studies involving PREO in LPS-stimulated RAW 264.7 cells demonstrated a similar trend of reduced oxidative stress and inflammatory responses [18]. Similarly, Taikong blue lavender essential oil (TLEO) was shown to decrease NO and ROS levels in LPS-induced HaCaT cells [26]. PREO also exerted anti-inflammatory effects by inhibiting the expressions of these pro-inflammatory factors (IL-1β, IL-6, IL-8, and TNF-α). These results are consistent with different research conducted on the individual essential oils. A study on LPS-induced human THP-1 cells exposed to three different Asian herb essential oils (*C. martini*, *T. Vulgaris*, and *P. aeruginosa*) showed limited effects on the secretion levels of TNF-α and IL-1β in LPS [27]. According to other research, ginger EO and *Piper nigrum Linn* EO can raise SOD activity in formalin-induced BALb/c mice [28,29]. However, PREO showed different results depending on the LPS induction time. A total of 0.1% PREO reduced the inflammation remarkably, even after 18 h of LPS exposure. PREO consistently showed a time-dose-dependent effect on LPS-induced HaCaT cells (see Supplementary Data: Figures S1 and S2). As overall 0.1% PREO showed the best outcome, and it was selected for the pathway study. The results showed that PREO could restrain the TLR4 pathway by downregulating the expression of MyD88 and TRIF, and then it decreased the activity of the NF-κB pathway to prevent inflammation and oxidative stress. These results matched our previous experiments of PREO on LPS-induced murine macrophage cells and DSS-induced mouse colitis. The observed anti-inflammatory effects of PREO are consistent with mechanisms elucidated for other phytochemicals. For instance, curcumin from *Curcuma longa* has been reported to concurrently inhibit NF-κB and MAPK signaling, leading to attenuated inflammatory responses, a mode of action comparable to that of PREO [30]. Similarly, apigenin, a flavonoid commonly found in celery, thyme, and basil, has been shown to suppress NF-κB and STAT3 activation in LPS-induced colonic epithelial carcinoma cells, further corroborating the potential of plant-derived compounds in modulating critical inflammatory pathways [31]. Moreover, plant essential oils are known to influence additional inflammatory pathways beyond NF-κB, including TLR, MAPK, NLRP3, and Nrf2/ARE cascades [32]. An herbal formulation containing rose hips, for example, has demonstrated inhibitory effects on the IRAK-1/TAK1 and TBK1/IRF3 pathways alongside suppression of NF-κB signaling [33,34]. In the present study, PREO was also found to regulate the transcriptional expression of p38 MAPK at the RNA level, indicating its capacity to regulate the MAPK pathway, a signaling system that integrates extracellular stimuli and orchestrates gene expression through kinases such as p38, JNK, and ERK. Although this study focused on p38 MAPK, it is plausible that PREO might also affect JNK and ERK pathways, which are frequently activated in tandem in inflammatory states. Collectively, these findings underscore the conserved efficacy of plant-based agents in targeting pivotal nodes within inflammatory cascades.

**Figure 8:**
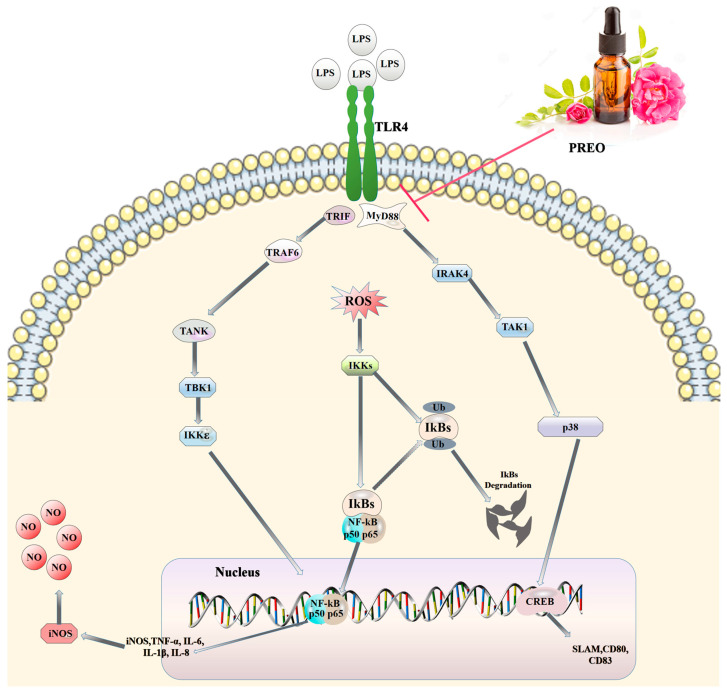
A proposed schematic presentation mechanism of PREO in inhibiting LPS-stimulated inflammation in HaCaT cells.

As PREO is enriched with versatile small compounds, its biofunctions may be determined by chemical composition. However, it may be hard to tell which is the most active one for bioactivities. From the GC–MS analysis of our previous results, 57 compounds were determined [18]. Most are terpenoids and monoterpenoids. The highest compound in amount is Citronellol with 54.37%. Citronellol is a natural monoterpenoid [16]. We therefore explored the potential interactions between Citronellol and the predicted network pharmacology targets using molecular docking analysis. Citronellol showed comparatively better interaction with CHUK and IKBKB proteins, which are the symbols of Ikk-α and IκB-α, respectively. The binding affinities were also considerable with the TLR4 adaptor protein MyD88 and the inflammatory enzyme PTGS2. Compared with the relative peak area percentage (54.37%), Citronellol may be the most responsible one for bioactivities, and TLR4-NF-κB possibly the most interactive pathway (Figure 8). Importantly, the convergence on Citronellol reflects both its chemical dominance and network ranking; however, the multicomponent nature of essential oils suggests that minor constituents and potential synergistic interactions may also contribute to the overall bioactivity. The anti-inflammatory and anti-oxidative properties of Citronellol have also been demonstrated by several studies [17]. Citronellol shows protective effects against rhabdomyolysis-induced acute kidney injury in mice by inhibiting NF-κB and IL-1β [35]. In DMBA-induced mammary carcinogenesis, Citronellol downregulates NF-κB and other inflammatory markers while increasing IL-10 levels [36]. Thus, this work extends our earlier findings in mouse macrophages by establishing PREO efficacy in a human keratinocyte inflammation model, which more directly reflects cutaneous inflammatory pathology.

To further contextualize the mechanistic interpretation, it is important to note that the present in silico findings provide supportive but not definitive evidence for direct ligand–target engagement. The molecular docking analysis was performed as an exploratory screening step and was not complemented by redocking validation, positive control benchmarking, or molecular dynamics simulations; therefore, the predicted binding energies should be interpreted qualitatively rather than as quantitative indicators of binding stability in a dynamic biological environment. In addition, although Citronellol was highlighted due to its high relative abundance and favorable docking profile, the multicomponent nature of PREO suggests that the observed bioactivity may arise from combined or synergistic effects among multiple constituents. Consequently, Citronellol should be considered a major candidate contributor rather than the sole active principle. The 200 ns MD simulations provide dynamic insights that complement the molecular docking analysis by capturing the temporal evolution of ligand–protein interactions in a solvated environment. While docking predicts a static binding pose, MD simulations allow assessment of the persistence and stability of these interactions under physiologically relevant conditions. The stable RMSD trajectories and low RMSF values within the binding regions suggest that Citronellol and geraniol can maintain consistent interactions with the predicted targets over extended simulation times. The favorable binding free energies estimated by MM-GBSA further support the stability of these complexes.

Collectively, these findings provide dynamic support for the docking predictions and suggest that major constituents of Pingyin rose essential oil may interact with inflammation-related targets stably. However, these computational observations should be interpreted as supportive evidence, and further experimental validation would be necessary to fully establish their biological relevance.

## 4. Materials and Methods

### 4.1. Chemical Reagents

PREO was donated by Jinan Wanfeng Rose Products Co., Ltd., Pingyin, Shandong Province, China. It was extracted via steam distillation from Rosa rugosa cv. Plena is cultivated in Shandong Province. The main chemical components of PREO were identified and analyzed by gas chromatography-mass spectrometry (GC–MS) in our preliminary work, and the relevant findings have been published in a previous study [18], where the corresponding table is cited herein; the full experimental data will be submitted as Supplemental S4. The chemical profile of the PREO used in this study was confirmed to be consistent with our previously reported GC–MS analysis. Sodium pyruvate was purchased from Solarbio Life Sciences (Beijing, China). Dulbecco’s Modified Eagle Medium (DMEM) and fetal bovine serum (FBS) were purchased from GIBCO BRL (Grand Island, NY, USA). The LPS (*Escherichia coli* O127:B8) and the 20,70-dichlorofluorescein-diacetate (DCFH_2_-DA) were bought from Sigma-Aldrich (St. Louis, MO, USA). MTT assay kit, Bicinchoninic (BCA) protein assay kit, total nitric oxide (NO) assay kit, superoxide dismutase (SOD) assay kit, and malondialdehyde (MDA) assay kit were obtained from Beyotime Institute of Biotechnology, Ltd. (Shanghai, China). Rabbit monoclonal antibodies against IκB-α, p-IκB-α, NF-κB, p-NF-κB, MyD88, TRIF, GAPDH, and mouse monoclonal antibody against β-actin were purchased from Cell Signaling Technology, Danvers, MA, USA (CST). The polymerase chain reaction (PCR) primers of β-actin, TNF-α, IL-1β, IL-6, and IL-8 were acquired from BGI (Beijing Genomics Institute), Beijing, China. RNA extraction kit was purchased from Transgen Biotech Co., Ltd. (Beijing, China). High-sig ECL Western Blotting Substrate was obtained from Tanon™ (Tanon, Shanghai, China). All other chemicals used in this study were of analytical grade and purchased from Beijing Chemical Works (Beijing, China). The PREO was dissolved in the serum-containing medium to achieve the final desired concentration.

### 4.2. Cell Culture

HaCaT cells were collected from the Stem Cell Bank, Institute of Zoology (China Academy of Sciences, Beijing, China). The cell was maintained in Dulbecco’s Modified Eagle Medium (DMEM) supplemented with 10% FBS, 1% glutamax, 1% Penicillin-Streptomycin (P/S), and 1% sodium pyruvate at 37 °C in 5% CO_2_ atmosphere. HaCaT cells were maintained in T-25 cm^2^ flasks (Corning Glass Works, Corning, NY, USA). The medium was refreshed every second day [26].

### 4.3. Construction of Inflammation Model and Cytotoxicity Assay

Cells were seeded in 96-well plates at the rate of 2 × 10^4^ cells/well. It was cultured up to 72 h to determine the cell growth curve. Several concentrations of LPS were used to construct an inflammation model by inducing oxidative stress in HaCaT cells. Cells were incubated with or without the addition of 2.5 µg/mL, 2 µg/mL, 1.5 µg/mL, or 1 µg/mL LPS for up to 20 h. The expressions of pro-inflammatory cytokines IL-8, IL-1β, and IκB-α for 6 h, 18 h, and 20 h were determined by RT-PCR. To determine its cytotoxicity, HaCaT cells were incubated with various concentrations of PREO (0.001–1% *v*/*v*) for 20 h.

### 4.4. Cell Viability Assay

The cytotoxicity of PREO and LPS against HaCaT cells was evaluated using the MTT assay. Cells were seeded in 96-well plates at 2 × 10^4^ cells per well and cultured. After a 24 h incubation period, cells were exposed to 2.5 μg/mL LPS for 20 h. Untreated cells served as the negative control to establish baseline viability. LPS-treated cells (2.5 µg/mL) acted as the positive control for inflammation induction. Then various doses of 0.001–0.1% (*v*/*v*) PREO were added for an additional 6–18 h using DMSO as a vehicle in negligible amounts. The MTT assay was performed to evaluate the cells’ viability [18].

%Cell Viability = (Absorbance of sample/Absorbance of control) × 100

### 4.5. Nitric Oxide (NO) Quantification and Measurement of ROS Production

HaCaT cells were seeded at 2 × 10^4^ cells/well in 96-well plates and incubated at 37 °C for 24 h. LPS (2.5 μg/mL) was added and incubated for another 20 h. After treating with various doses of PREO for an additional 12 h and 18 h, cells were subjected to NO level evaluation. Nitrite was measured using Griess reagents to give an estimation of NO production. Briefly, 100 μL of the cell supernatant was mixed with 100 μL of Griess reagents and incubated at room temperature for 10 min. Absorbance was determined at 540 nm using a spectrophotometer (TECAN, Männedorf, Switzerland).

For ROS measurement, the HaCaT cells (2 × 10^4^ cells per well in a 96-well plate) were exposed to the previous series of treatment with LPS and PREO. Then, following the collection, they were washed with PBS and incubated with 10 μM DCFH2-DA (dissolved in PBS). After incubation at 37 °C for 30 min, DCFH2-DA was removed, and HaCaT cells were washed in PBS again. Intracellular ROS, as indicated by DCF fluorescence, was observed with a fluorescence microscope. The fluorescence intensity was quantified using a spectrophotometer at the excitation of 480 nm and emission of 520 nm [18].

### 4.6. Determination of MDA and SOD Level in LPS-Induced HaCat Cells

LPS-induced HaCaT cells (1 × 10^4^ per well in a petri dish), treated with different doses of PREO, were washed twice with cold PBS and harvested with a cell scraper. Then cell lysis fluid was added to lyse cells, and the supernatant from centrifugation of cells (10,000× *g* at 4 °C for 5 min) was collected. The level of MDA and the activity of SOD were determined by following the manufacturer’s instructions for commercial kits.

### 4.7. Quantitative Real-Time Polymerase Chain Reaction (qRT-PCR)

Following the manufacturer’s instructions, the total RNA from the HaCaT cells (5 × 10^6^ cells per well in a petri dish treated with LPS and PREO) was isolated using a Trizol reagent (TransGen Biotech, Beijing, China). The concentration and integrity of the RNA were measured at a 260/280 nm ratio. A total of 0.5 μg of RNA was reverse-transcribed using the Prime Script RT reagent ki t(TransGen Biotech, Beijing, China) for the cDNA synthesis. The PCR primers were designed using NCBI, and the primer sequences have been presented in Table 3. As an invariant housekeeping gene internal control, the β-actin gene was used. Briefly, the reaction series was as follows: 50 °C for 2 min, 95 °C for 30 s for one cycle; then 95 °C for 5 s, 59 °C for 15 s, and 72 °C for 45 s for 40 cycles. The relative gene expression was quantified by the comparative 2^−∆∆CT^ method [26]. All the reactions were conducted in triplicate.

### 4.8. Western Blotting Analysis

After treatment, the HaCaT cells (5 × 10^6^ cells per well in a petri dish) were collected, and the total protein was extracted using cell lysis buffer for Western blot containing protease inhibitors or phosphatase inhibitors. After 30 min on ice, the solution was centrifuged for 15 min at 4 °C at 3000 rpm. Following the microplate process, the supernatants were separated, and protein quantities were measured using the BCA protein assay. Each sample was split into 30 µg of proteins using sodium dodecyl sulfate-polyacrylamide gel electrophoresis (SDS-PAGE), and the proteins were subsequently transferred to polyvinylidene difluoride membranes (PVC) from Merck KGaA in Darmstadt, Germany. When using 5% non-fat milk in tris-buffer saline (TBST), the membrane was blocked at 4 °C. After that, the membrane was incubated with primary antibodies (1:1000, *v*/*v*) and three TBST washes. Following that, the membrane was incubated at room temperature for two hours with secondary antibodies (1:1000, *v*/*v*). Tanon™ High-sig ECL Western Blotting Substrate (Tanon, Shanghai, China) was applied to the membrane to visualize the band density as per manufactures instruction. Finally, using TanonImage (v1.00, Shanghai, China) image processing software (ImageJ version 1.54r, National Institutes of Health, Bethesda, MD, USA), bands were quantified as per their density and evaluated.

### 4.9. Network Pharmacology Study

#### 4.9.1. Screening and Acquisition of Components-Targets for PREO Components

Each compound was searched in TCM for the System Pharmacology database and analysis platform (TCMSP, https://tcmspw.com/tcmsp.php accessed at 10 September 2022) to find out its oral bioavailability (OB). OB is an essential marker to determine the nature of classified drugs, focusing on unchanged drug percentages to reach the circulation system and referring to the identification of the dose range [37]. The Swiss Target prediction (http://www.swisstargetprediction.ch/) web tool was used to predict potential targets for selected compounds (CT) under “*Homo sapiens*”. Using the keywords “Skin inflammation” (SI) and “Skin oxidative stress” (SOS), related genes were downloaded from the GeneCards platform (https://www.genecards.org/).

#### 4.9.2. Construction and Analysis of Protein-Protein Interaction Network (PPI)

Venny 2.1.0 was used to find out common targets from the potential targets. The common targets were placed in the STRING database to determine the possible PPI [38]. Cytoscape 3.9.1v was used to construct a “compound-target network” using those common targets [39].

#### 4.9.3. Molecular Docking

The sequence of the top 5 proteins was retrieved from the UniProtKB database (https://www.uniprot.org/). Based on the BLAST result of PSI-BLAST (https://blast.ncbi.nlm.nih.gov), the best templates were selected according to the percentage of sequence coverage, E-value, species, and percentage of sequence similarity. PDB (protein databank; https://www.rcsb.org) was used to download the best template. The most active component of PREO, based on GC–MS percentage and network pharmacology, was selected [18]. The structures of the compound were downloaded in SDF format from Pubchem and were prepared as ligands for docking (https://pubchem.ncbi.nlm.nih.gov). Before docking analysis, both ligands and receptors were optimized by Autodock Tool software (ADT4, v4.2.6). Ligand binding sites for non-enzyme proteins were predicted by CastP [40]. Allosteric sites for enzymes were predicted by Passer 2.0. Then AutoDoc Vina (v1.1.2) was used to perform molecular docking [41].

#### 4.9.4. Molecular Dynamic Simulation

Molecular dynamics (MD) simulations were conducted to investigate the stability and dynamic behavior of the interactions between the principal PREO constituents, Citronellol and geraniol, and their predicted protein targets. The initial coordinates for the complexes were obtained from the highest-ranked docking conformations. Each system was solvated in an explicit TIP3P water box with a 10 Å buffer and neutralized by the addition of Na^+^ or Cl^−^ counterions. Protein parameters were assigned using the AMBER ff14SB force field, while ligand parameters were generated with the General Amber Force Field (GAFF2). Following energy minimization (5000 steps), the systems were equilibrated under NVT and NPT ensembles for 100 ps each at 300 K and 1 bar. A 200 ns production simulation was then performed with a 2 fs integration time step, and long-range electrostatic interactions were treated using the Particle Mesh Ewald method. Trajectory analyses included root mean square deviation (RMSD), root mean square fluctuation (RMSF), and radius of gyration (Rg) to evaluate structural stability and flexibility, while binding free energies were estimated using the MM-GBSA method.

### 4.10. Statistical Analysis

All values were expressed as mean ± SD of three parallel measurements, and the analysis was carried out in triplicate. Statistical analysis was carried out by a one-way analysis of variance (ANOVA) test using a statistical package program (SPSS 13.0) and the significance of the difference between means was determined by Duncan’s multiple range test at (*p* < 0.05) a significant level.

## 5. Conclusions

Skin inflammation can be life-threatening; consequently, as skin is the primary defense interface of our body against the outside environment. However, PREO is a potential anti-inflammatory agent for the treatment of skin-related inflammations. This study demonstrates that PREO, rich in Citronellol (54.37%), exhibits potent anti-inflammatory and antioxidant effects in LPS-induced HaCaT cells. PREO significantly reduced oxidative stress markers (NO, ROS, MDA) while enhancing SOD activity. Mechanistically, PREO suppressed the TLR4-NF-κB pathway by downregulating MyD88 and TRIF expression, inhibiting NF-κB p65 and IκB-α phosphorylation, and attenuating pro-inflammatory cytokine production (TNF-α, IL-1β, IL-6, IL-8). Molecular docking highlighted Citronellol’s strong binding affinity with key targets (IKKβ, MyD88), suggesting its pivotal role in mediating these effects. These findings validate the traditional use of PREO and position it as a promising candidate for managing skin inflammation.

Despite the promising anti-inflammatory and antioxidant effects observed in this study, several considerations should be acknowledged to strengthen the translational relevance of PREO. First, although the HaCaT model provides valuable mechanistic insight, it does not fully recapitulate the complexity of human skin physiology, including immune–epidermal interactions and barrier function. Second, while Citronellol was identified as the major active component through network pharmacology and molecular docking, the potential synergistic or antagonistic effects among the multiple constituents of PREO remain unclear. Third, the reliance on mRNA and selected protein markers limits comprehensive pathway resolution, and additional upstream or parallel signaling cascades (e.g., JNK/ERK branches of MAPK or Nrf2-mediated antioxidant responses) may also contribute to the observed bioactivity. Future studies integrating multi-omics approaches, standardized oil characterization, and advanced skin models (e.g., 3D reconstructed epidermis) would further clarify the mechanistic landscape and enhance the clinical translation potential of PREO-based formulations.

## Figures and Tables

**Table 1:** Active ingredients of PREO depending on OB%.

Name	PubChem ID	Molecular Formula	Molecular Weight (g/mol)	OB%
Methyl Eugenol	7127	C_11_H_14_O_2_	178.25	73.36
Citronellol	8842	C_10_H_20_O	156.3	38.05
Ethyl octanoate	7799	C_10_H_20_O_2_	172.3	33.05
Farnesol	445,070	C_15_H_26_O	222.4	28.44
Geranyl Acetate	1,549,026	C_12_H_20_O_2_	196.32	25.4

**Table 2:** Molecular docking values with Citronellol and pathway proteins.

Protein	PDB ID_Chain	Affinity	H/Alkyl/Pi Bonds	Van Dar Wales
CHUK	5EBZ_A	−6.4	Phe9; Trp15; Tyr32	Ala11; Gly13; Pro14; Glu16; Ile43; Val79; Pro80; Glu82; Leu92
IKBKB	4KIK_A	−5.7	Val29; Lys44; Met96; Glu97; Cys99; Ile165	Leu21; Ala42; Val74; Tyr98; Val152; Asp166
MAPK14	1A9U_A	−4.3	Val239; Leu246; Lys267; Val290; Leu291	Gly240; Thr241; Pro242; Gly243; Leu289; Asp292
MyD88	4DOM_A	−4.5	Tyr167; Pro169; Ile172; Asp195; Val198	Cys168; Val193; Ser194; Glu232
PTGS2	5F19_A	−4.8	Pro41; Cys41; Cys47; Leu152	His39; Gln42; Asn43; Arg44; Gly45; Pro153; Gln46; Glu465; Lys468; Arg469

**Table 3:** Primers for RT-PCR analysis of relative genes.

Name	Forward	Reverse
*β-actin*	CCTAGAAGCATTTGCGGTGCACGATG	TCATGAAGTGTGACGTTGACATCCGT
*IL-6*	AAGTGCATCATCGTTGTTCATACA	GAGGATACCACTCCCAACAGACC
*IL-1β*	GTGCTGCCTAATGTCCCCTTGAAT	TGCAGAGTTCCCCAACTGGTACAT
*TNF-α*	TACAGGCTTGTCACTCGAATT	ATGAGCACAGAAAGCATGATC
*IκBα*	AACCTGCAGCAGACTCCACT	ACACCAGGTCAGGATTTTGC
*IL-8*	CTGATTTCTGCAGCTCTGTG	GGGTGGAAAGGTTTGGAGTATG
*MyD88*	TGCTCGAGCTGCTTACCAAG	CATCCGGCGGCACCAATG
*IRAK-4*	TCATGGCTGTTTCTGGCTGT	CCCAGATACAACCCCGCAAT
*IKKβ*	GTGGTTGTCCTCTTTTCGGC	AAGCTCACAGCCCTTAGCC
*TKB1*	GAGGAGGCCGCGGGA	AGAACCTGAAGACCCCGAGA
*TAK1*	CTTGCAGACTGGTCCTCTGG	TGGCGCCAAATCCTGAGGTAA
*IKKΣ*	AGAGGTACTCCTGGTGTCGG	GAGTGTGGGAAATCCGGAGA
*P38MAPK*	ATCCTCAGGCATGGAACGTG	ACTCCTTTGAGCCGTTTGGA
*TRIF*	CTGAGTGGTCTATGGCGTCC	TTGGAAATCAGCCAGTCCCC
*Caspase-8*	GCTCTTCAAAGGTCGTGGTCA	CTGAGCTGGTCTGAAGGCTGG

## Data Availability

The original contributions presented in this study are included in the article/Supplementary Material. Further inquiries can be directed to the corresponding author.

## Supplementary Materials

The following supporting information can be downloaded at: https://www.mdpi.com/article/10.3390/ijms27073174/s1.
