# Supplementary material for: Anti-Inflammatory Effects of Pingyin Rose Essential Oil in LPS-Induced HaCaT Cells: An in Vitro and in Silico Study
Source: Int J Mol Sci. 2026 Mar 31;27(7):3174. doi: 10.3390/ijms27073174 (PMC13072962; doi:10.3390/ijms27073174)
Supplement: Supplementary file 1 [file ijms-27-03174-s001.zip › supplemental-S1.pdf]

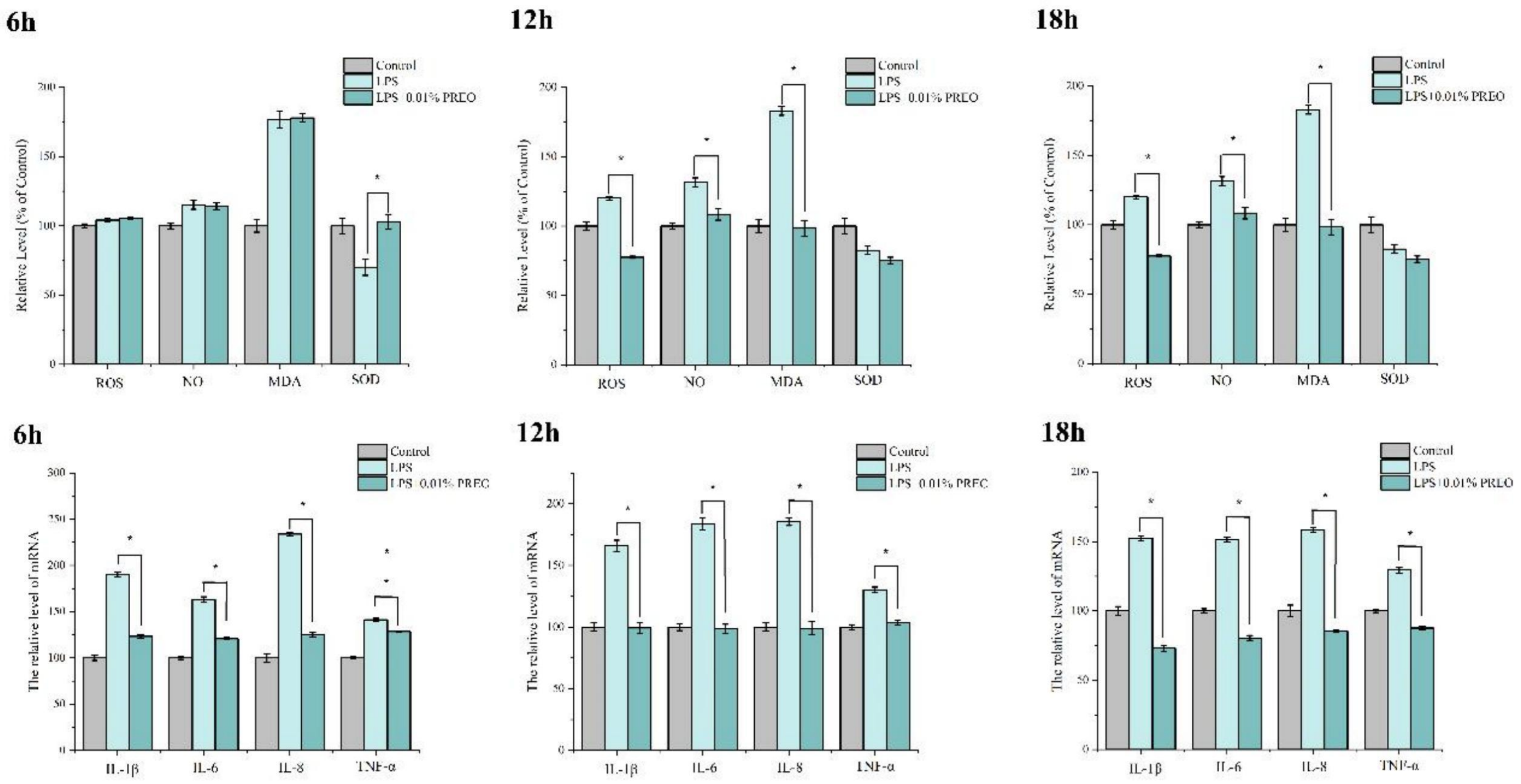

Figure S1: Comparative bar diagram of oxidative stress marker NO, ROS, SOD, MDA and inflammatory cytokines IL-1β, IL-6, IL-8. TNF-α at 6, 12 and 18 h exposure of PREO. The asterisk “*” defines the significance.
