# Supplementary material for: Anti-Inflammatory Effects of Pingyin Rose Essential Oil in LPS-Induced HaCaT Cells: An in Vitro and in Silico Study
Source: Int J Mol Sci. 2026 Mar 31;27(7):3174. doi: 10.3390/ijms27073174 (PMC13072962; doi:10.3390/ijms27073174)
Supplement: Supplementary file 1 [file ijms-27-03174-s001.zip › supplemental-S2.pdf]

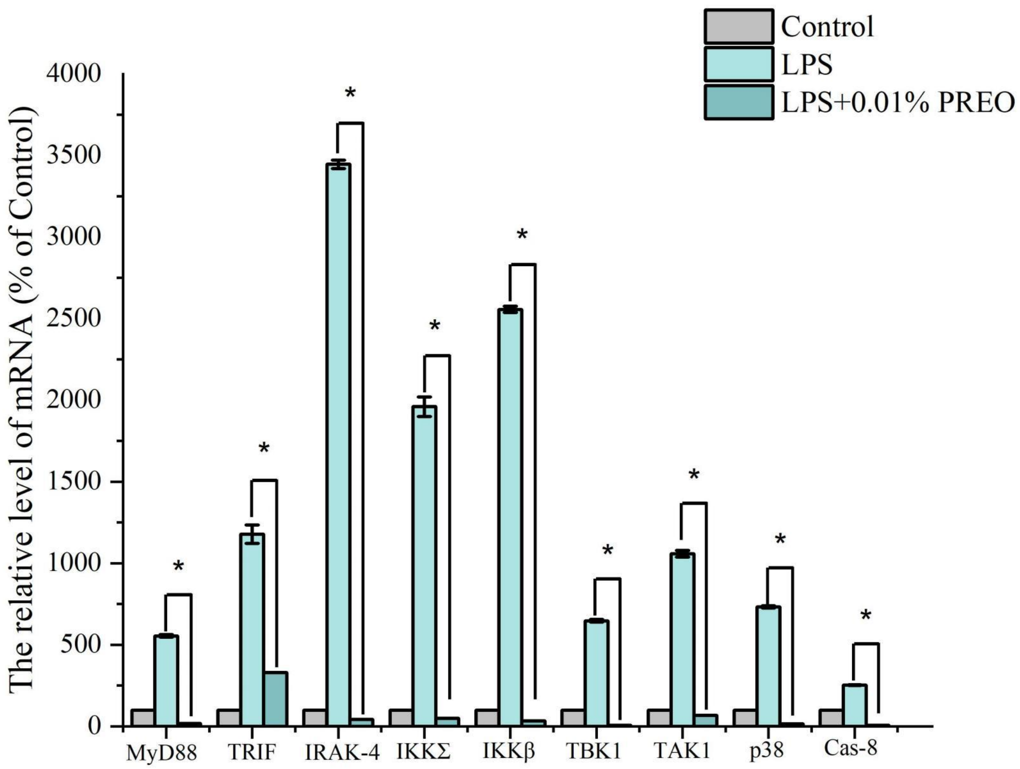

Figure S2: The effects of 0.01% PREO at 18 h on mRNA levels of TRL4 pathway.Proteins in HaCaT cells with or without LPS. The asterisk "*" defines the significance.
