# Supplementary material for: Anti-Inflammatory Effects of Pingyin Rose Essential Oil in LPS-Induced HaCaT Cells: An in Vitro and in Silico Study
Source: Int J Mol Sci. 2026 Mar 31;27(7):3174. doi: 10.3390/ijms27073174 (PMC13072962; doi:10.3390/ijms27073174)
Supplement: Supplementary file 1 [file ijms-27-03174-s001.zip › supplemental-S3.pdf]

Supplemental S3：Statement on Western Blot Experimental Design and Data Presentation

The segmentation of Western blot membranes in our supplementary files was necessitated by practical constraints—specifically, the high cost and limited availability of certain primary antibodies. To optimize antibody incubation conditions while conserving reagents, we followed a well-established practice of trimming PVDF membranes into strips based on the molecular weights of target proteins. This approach can enhance antibody specificity and reduce experimental costs without compromising data integrity.

In our initial submission, we included all original, uncropped images of the membrane strips in the supplementary materials. To ensure data integrity, we carefully realigned all strips derived from the same gel to reconstruct the complete membrane. As demonstrated, the strips—when arranged sequentially according to their numbering—precisely reassemble into a continuous gel, with consistent spacing between both molecular weight markers and sample lanes. This confirms their shared origin. We sincerely apologize that we are unable to provide an image of the intact membrane prior to sectioning, as the strips were already cut at the time of imaging.

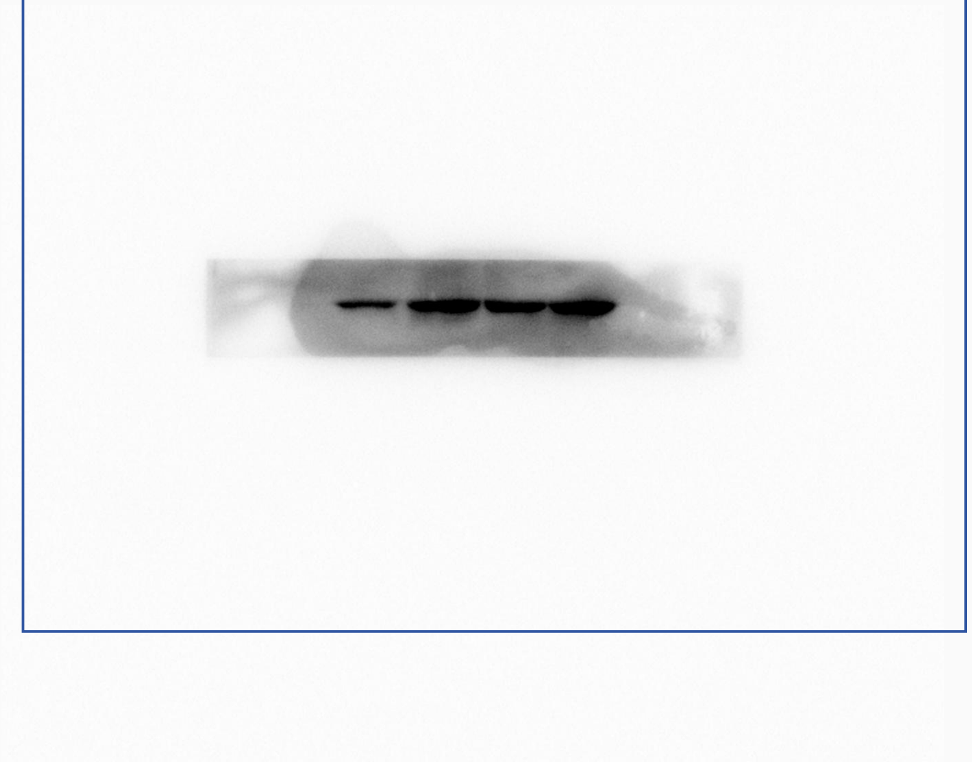

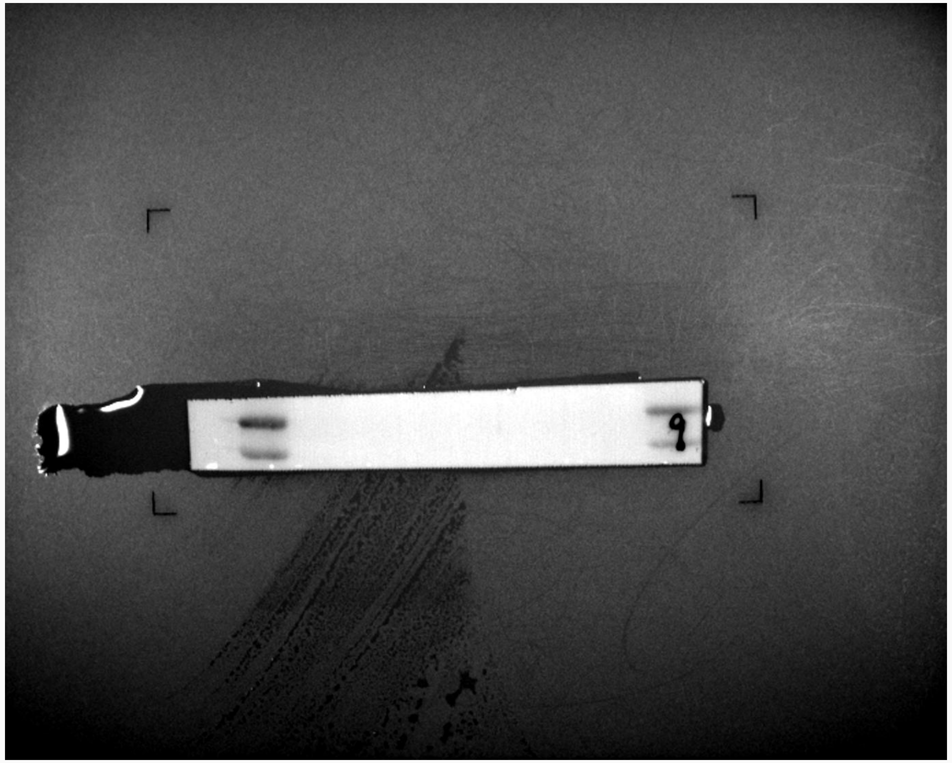
9

P65
IκB-α

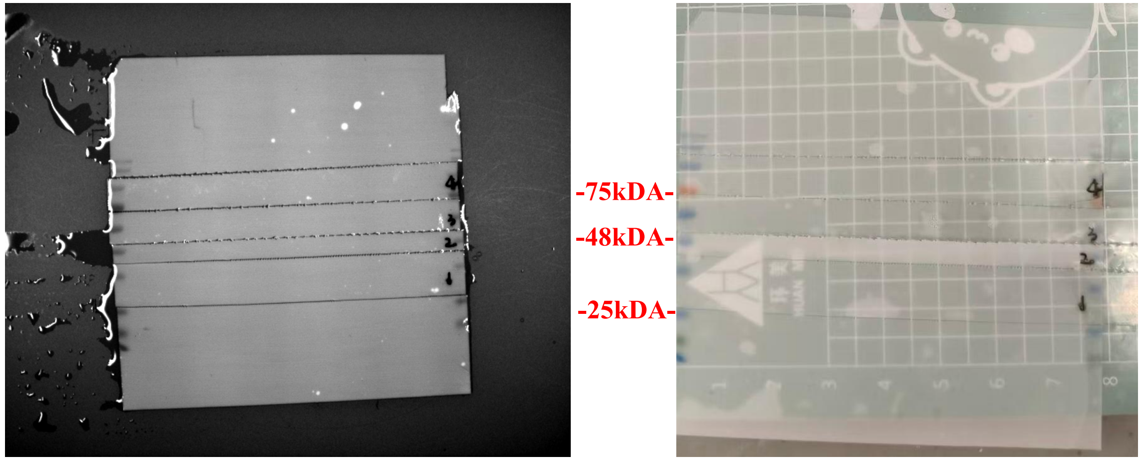

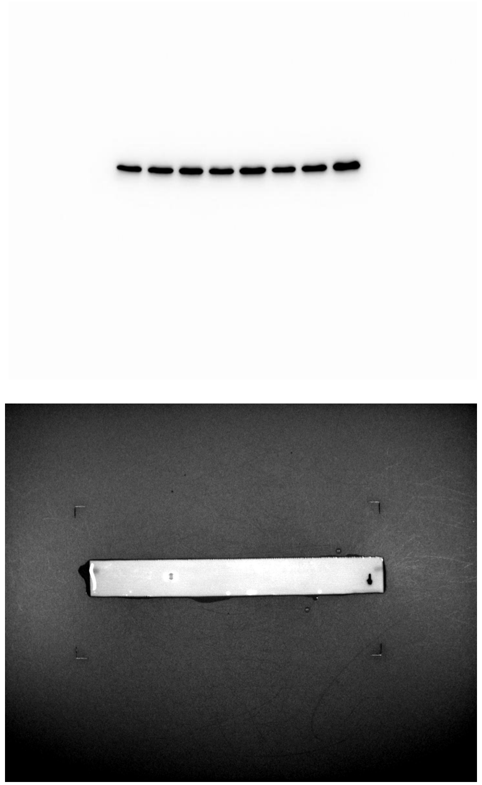

IκB-α (37 kDA)

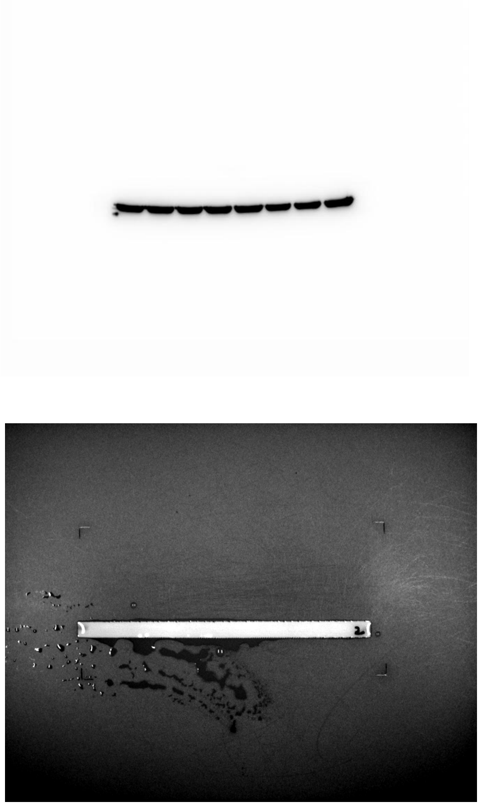

β-actin (42 kDA)

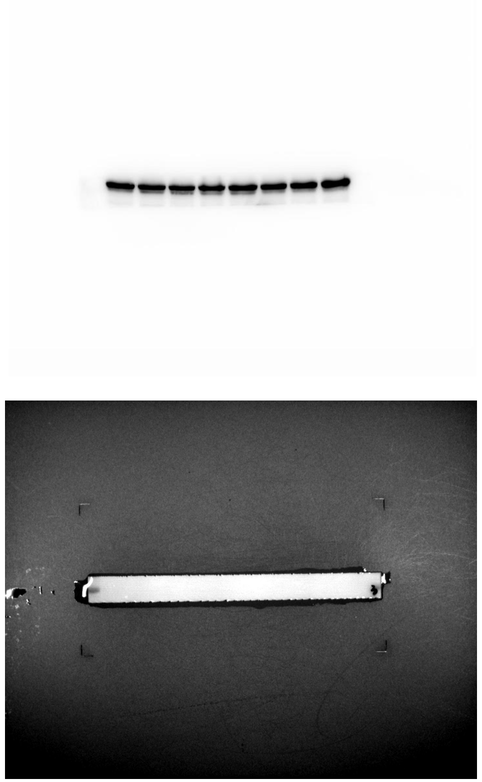

P65 (65 kDA)

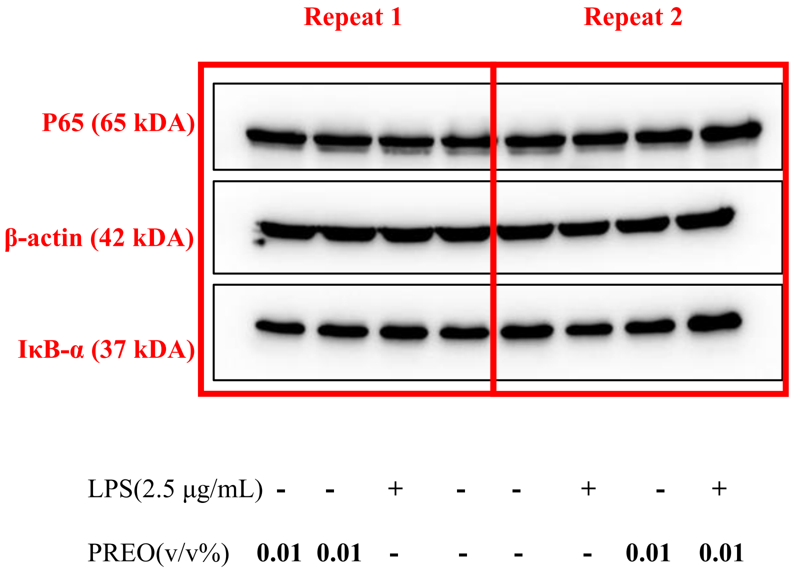

P65
IκB-α

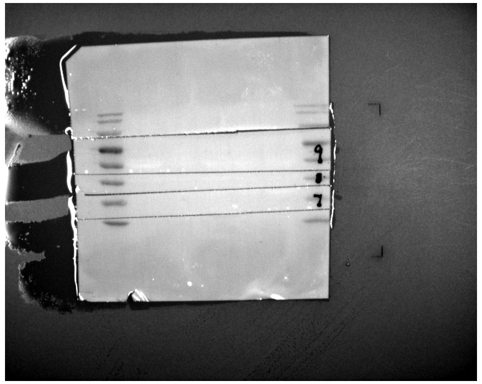

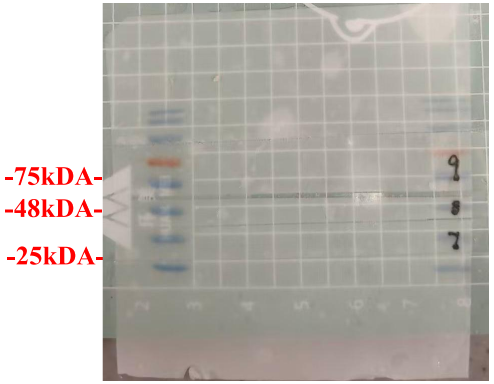

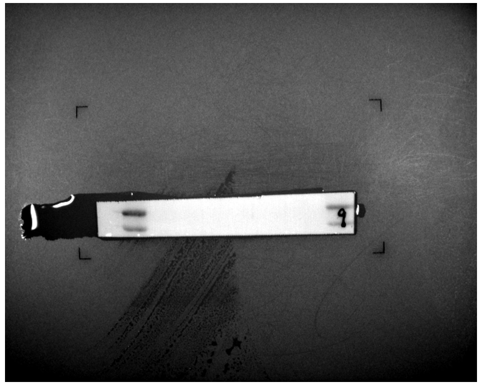

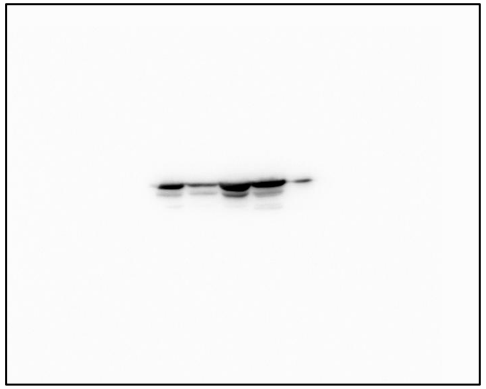

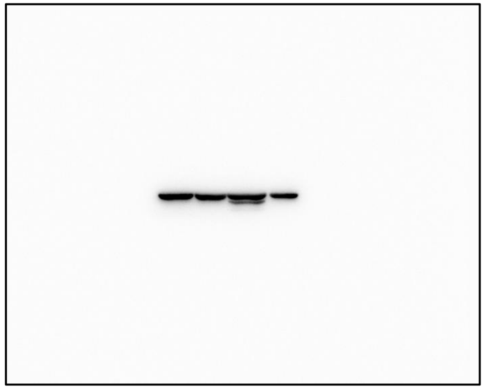

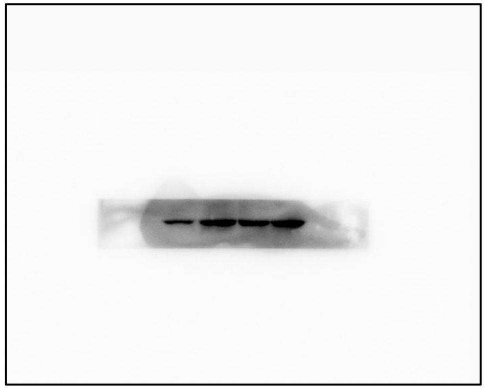

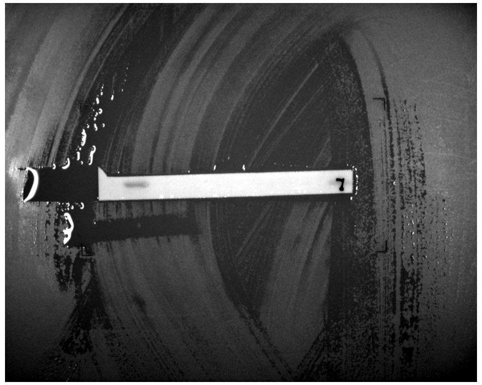

IκB-α (37 kDA)

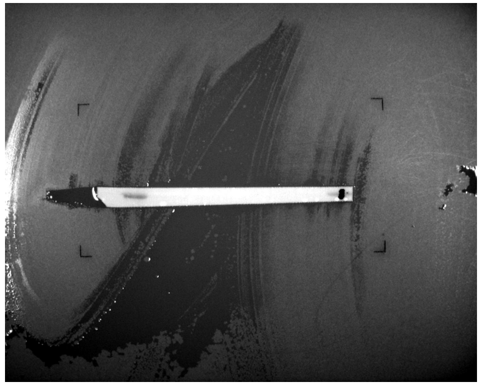

β-actin (42 kDA)

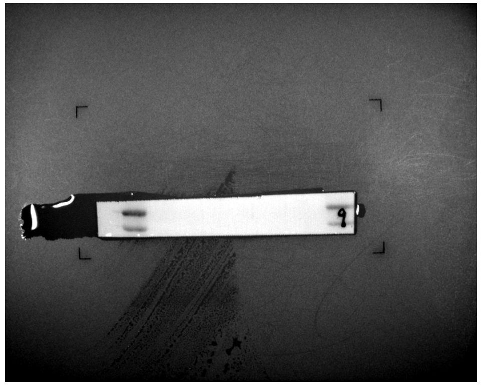

P65 (65 kDA)

Repeat 3

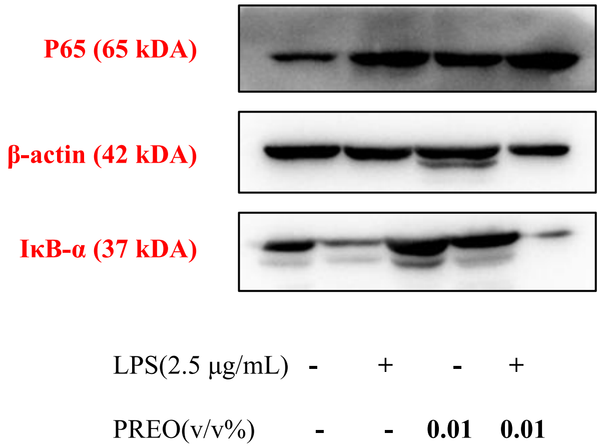

p-P65
p-IκB-α

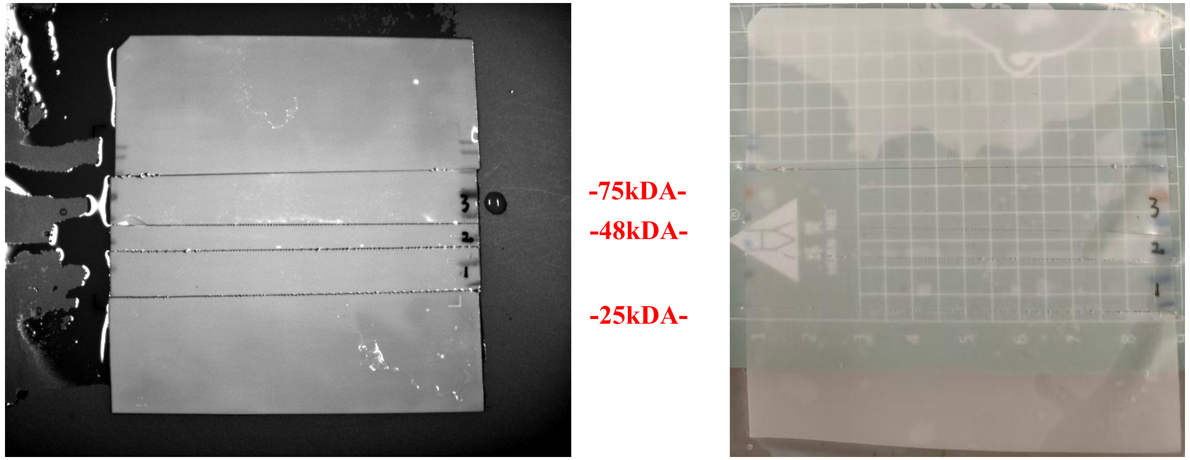

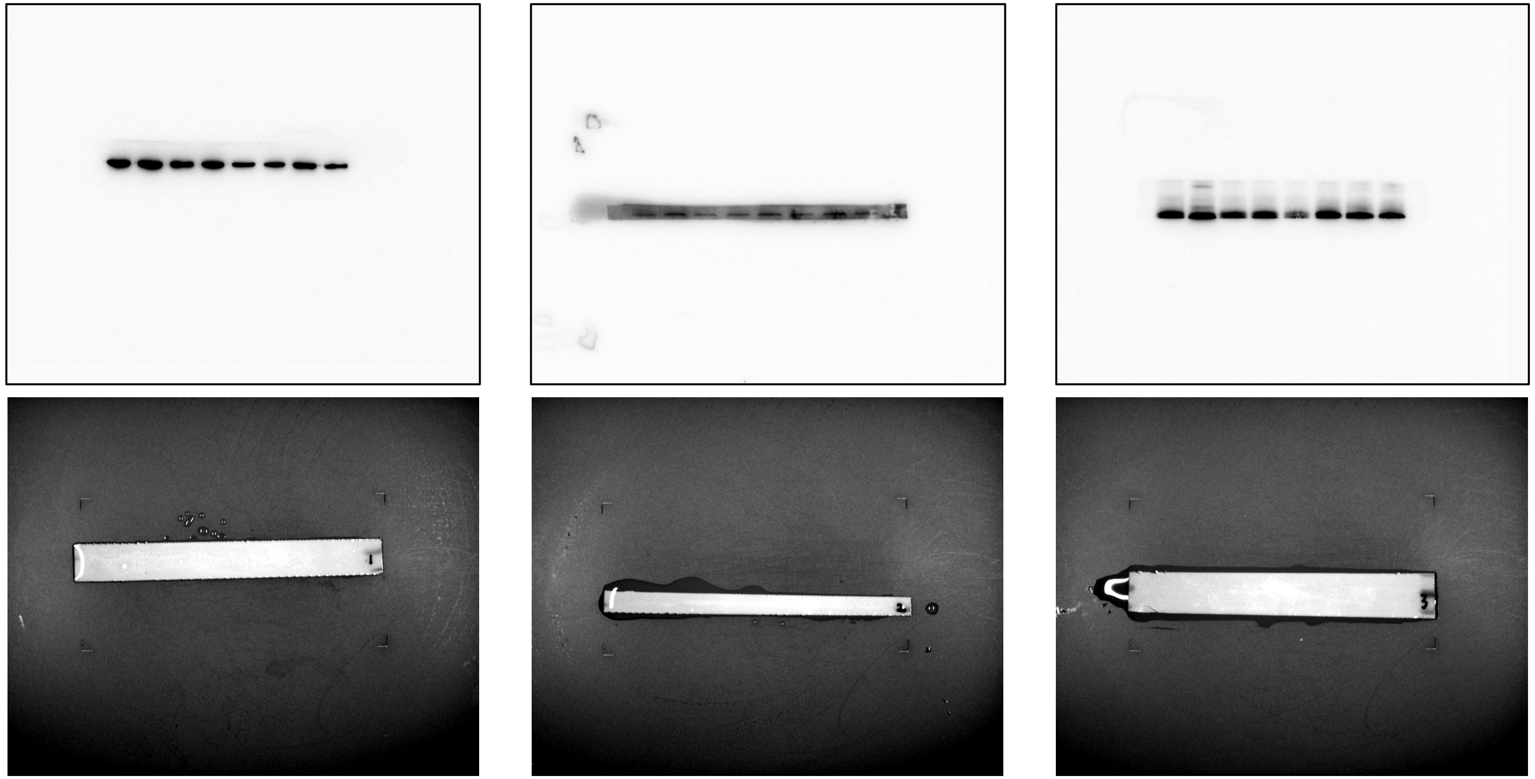

p-IκB-α (37 kDA)   β-actin (42 kDA)   p-P65 (65 kDA)

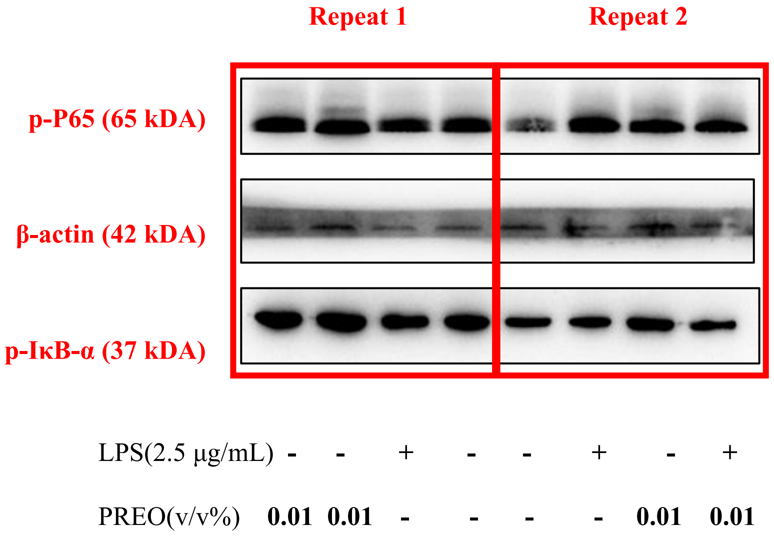

p-P65
p-IκB-α

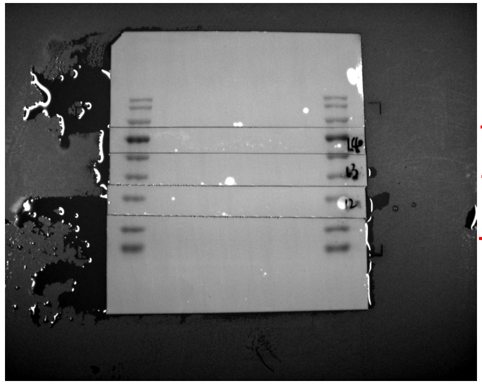

-75kDA-
-48kDA-
-25kDA-

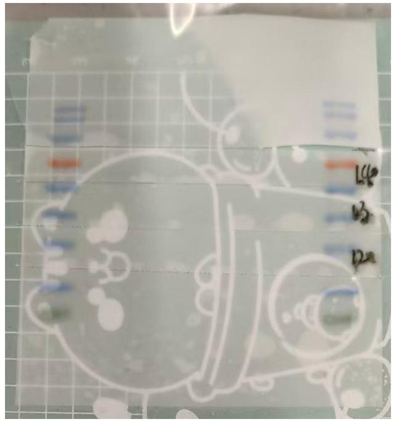

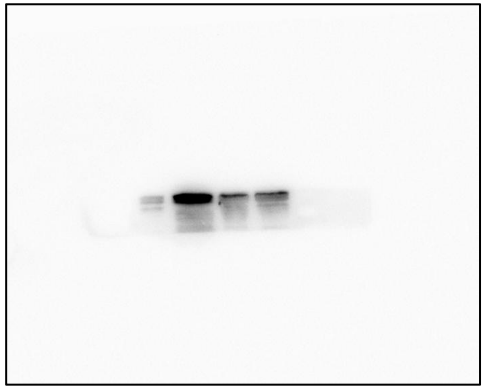

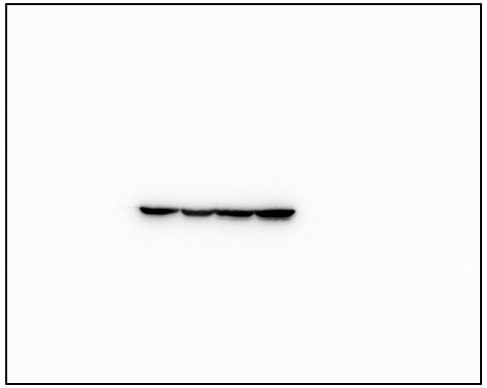

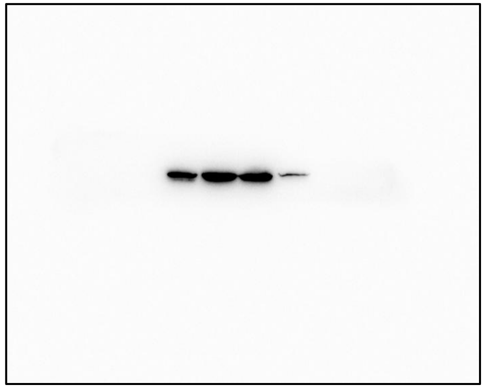

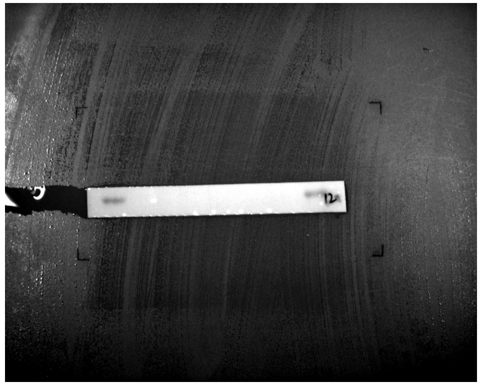

p-IκB-α (37 kDA)

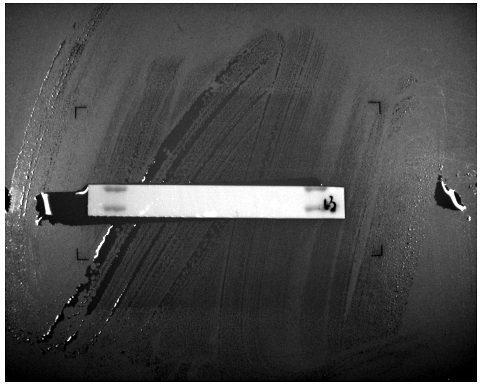

β-actin (42 kDA)

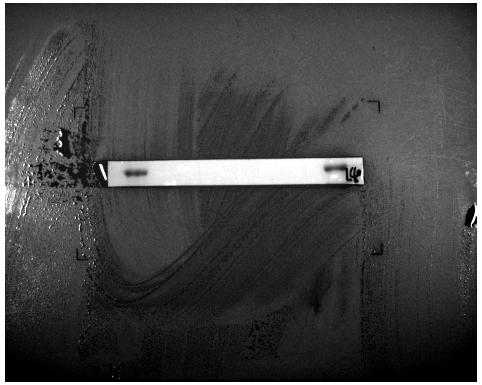

p-P65 (65 kDA)

Repeat 3

p-P65 (65 kDA)
β-actin (42 kDA)
p-IκB-α (37 kDA)

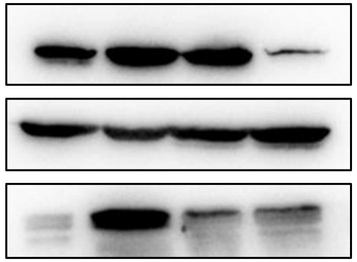

LPS(2.5 μg/mL)	-	+	-	+
PREO(v/v%)	-	-	0.01	0.01

TRIF
MyD88

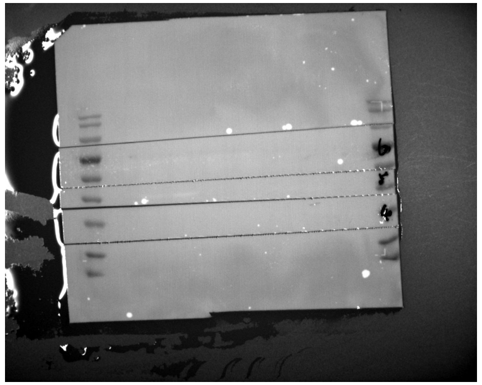

-75kDA-
-48kDA-
-25kDA-

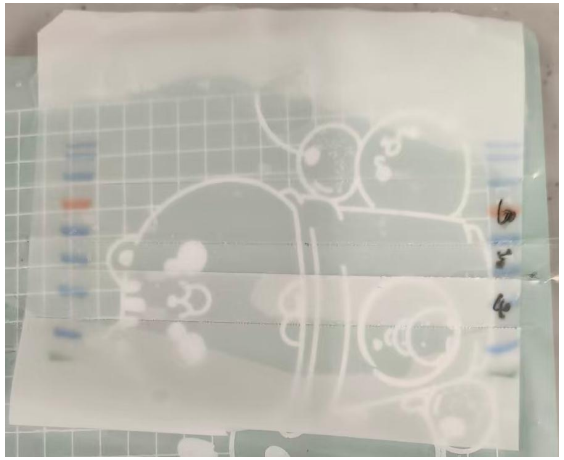

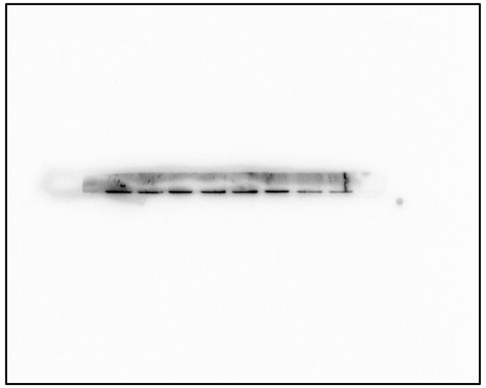

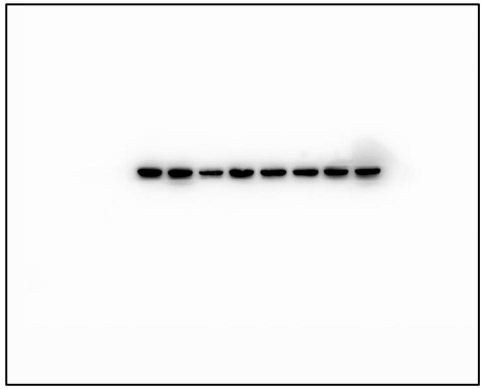

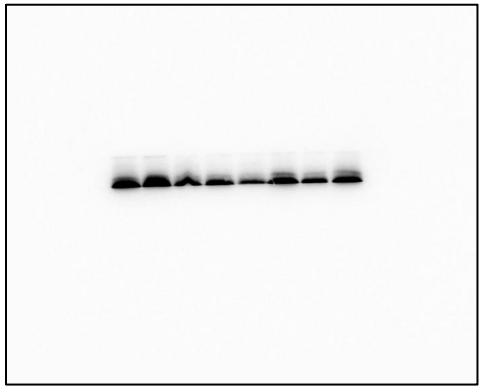

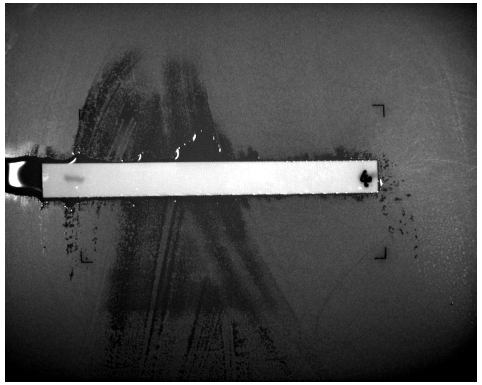

MyD88 (33 kDA)

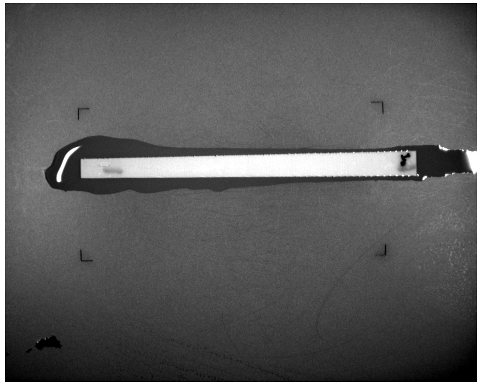

β-actin (42 kDA)

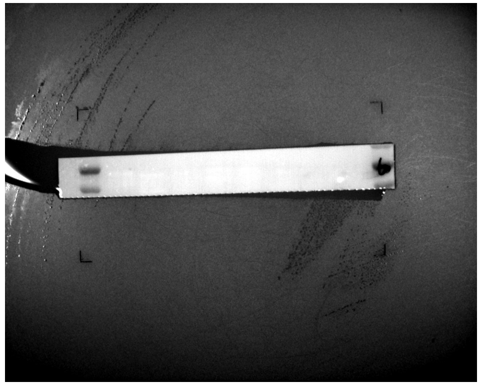

TRIF (70 kDA)

Repeat 1 Repeat 2

TRIF (70 kDA)
β-actin (42 kDA)
MyD88 (33 kDA)

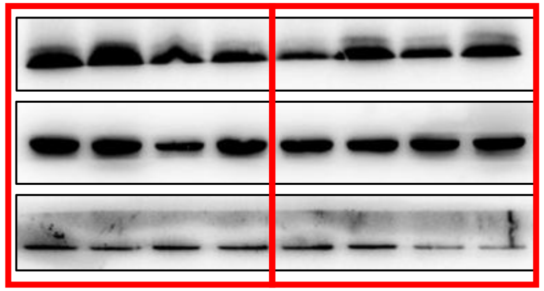

LPS(2.5 μg/mL)	-	-	+	-	-	+	-	+
PREO(v/v%)	0.01	0.01	-	-	-	-	0.01	0.01

TRIF
MyD88

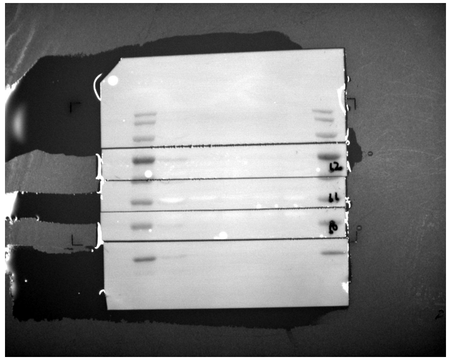

-75kDA-
-48kDA-
-25kDA-

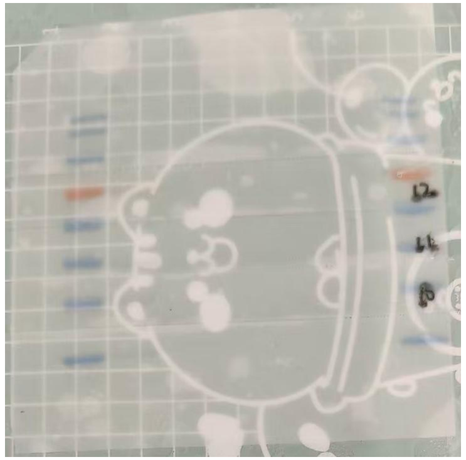

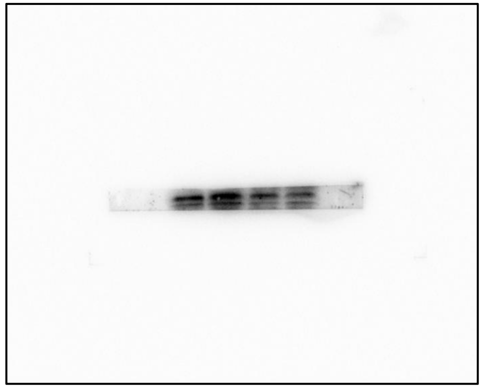

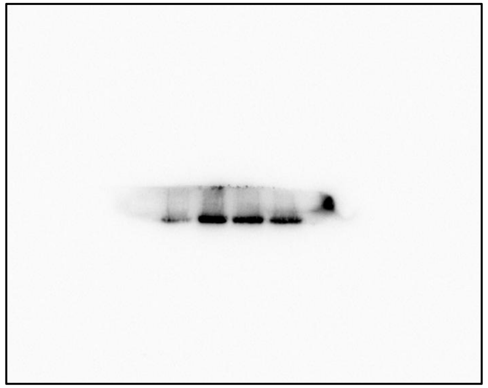

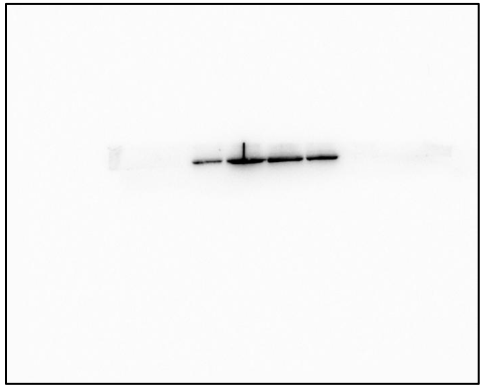

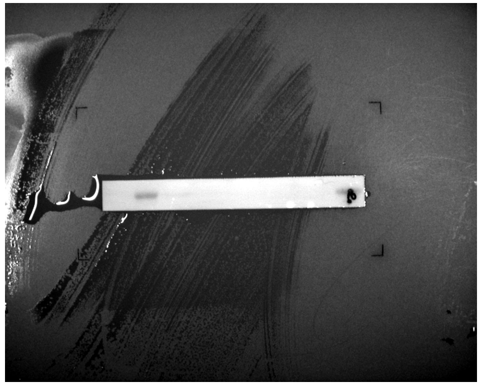

MyD88 (33 kDA)

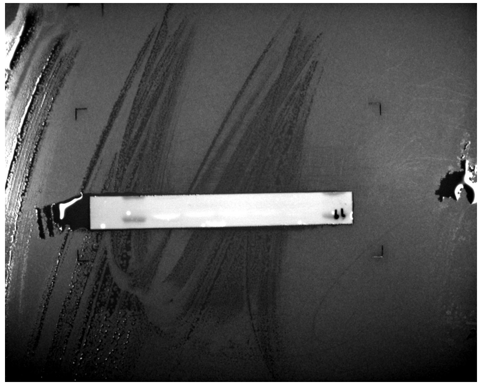

β-actin (42 kDA)

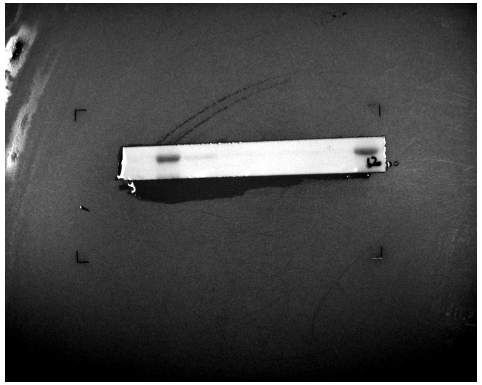

TRIF (70 kDA)

Repeat 3

TRIF (70 kDA)
β-actin (42 kDA)
MyD88 (33 kDA)

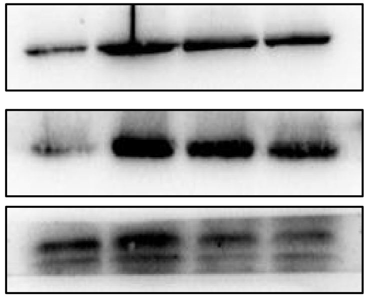

LPS(2.5 μg/mL)	-	+	-	+
PREO(v/v%)	-	-	0.01	0.01
