# Supplementary material for: Anti-Inflammatory Effects of Pingyin Rose Essential Oil in LPS-Induced HaCaT Cells: An in Vitro and in Silico Study
Source: Int J Mol Sci. 2026 Mar 31;27(7):3174. doi: 10.3390/ijms27073174 (PMC13072962; doi:10.3390/ijms27073174)
Supplement: Supplementary file 1 [file ijms-27-03174-s001.zip › supplemental-S4.pdf]

Supplemental S4.Main compounds detected in PREO through GC-MS analysis

No	RI	Compounds	Class	Relative peak area (%)
1	1028	α-Pinene	Monoterpene	0.73
2	1112	β-Pinene	Monoterpene	0.10
3	1124	2,4(10)-Thujadiene	Monoterpene	0.02
4	1161	β-Myrcene	Monoterpene	0.06
5	1199	L-Limonene	Monoterpene	0.13
6	1275	o-Cymene	Monoterpene	0.04
7	1280	Terpinolene	Monoterpene	0.05
8	1365	Rose oxide	Monoterpene	0.91
9	1367	trans-Rose oxide	Monoterpene	0.32
10	1429	Perillen	Monoterpene	0.02
11	1444	p-Cymenene	Monoterpene	0.02
12	1753	Geranyl acetate	Monoterpene	1.00
13	2169	Eugenol	Monoterpene	1.40
14	1231	2-Pentylfuran	Heteroaromatic	0.03
15	1282	trans-2-(2-Pentenyl) furan	Heteroaromatic	0.02
16	1413	Rosefuran	Heteroaromatic	0.01
17	1213	Eucalyptol	Monoterpenoid	0.02
18	1250	trans-β-Ocimene	Monoterpenoid	0.07
19	1252	β-Ocimene	Monoterpenoid	0.07
20	1469	Nerol oxide	Monoterpenoid	0.05
21	1514	Cyclohexane	Monoterpenoid	0.24
22	1547	β-Linalool	Monoterpenoid	1.36
23	1765	Citronellol	Monoterpenoid	54.37
24	1797	Nerol	Monoterpenoid	4.20
25	1847	Geraniol	Monoterpenoid	9.26
26	1495	Daucene	Sesquiterpene	0.20
27	1579	trans-α-Bergamotene	Sesquiterpene	0.28
28	1586	β-Copaene	Sesquiterpene	0.21
29	1661	α-Himachalene	Sesquiterpene	0.58
30	1727	β-Bisabolene	Sesquiterpene	0.17
31	1735	Bicyclogermacren	Sesquiterpene	0.85

No	RI	Compounds	Class	Relative peak area (%)
32	1746	α-Farnesene	Sesquiterpene	2.01
33	2215	α-Bisabolol	Sesquiterpene	0.67
34	2350	Farnesol	Sesquiterpene	0.40
35	1320	2-Heptanol	Fatty alcohols	0.05
36	1450	1-Octene-3-ol	Fatty alcohols	0.01
37	1615	Citronellyl formate	Fatty alcohols	0.15
38	1660	Citronellol acetate	Fatty alcohols	3.35
39	1903	2-Tridecanol	Fatty alcohols	0.15
40	2165	1-Tetradecanol	Fatty alcohols	0.14
41	1338	5-Hepten-2-one	Ketone	0.02
42	1465	6-Methyl-5-hepten-2-ol	Ketone	0.01
43	1598	Methyl nonyl ketone	Ketone	0.26
44	1809	Tridecanone	Ketone	1.85
45	2123	Cyclododecanol	Ketone	0.07
46	2217	Pentacosane	Ketone	4.66
47	1435	Ethyl octanoate	Ester	0.02
48	1813	Phenethyl acetate	Ester	0.24
49	2098	Tetradecanol acetate	Ester	0.56
50	2100	Heneicosane	Alkane	2.26
51	2108	Pentadecanol	Alkane	0.04
52	1184	Heptanal	Aldehydes	0.12
53	1639	Isosativene	Terpene	0.11
54	1725	Naphthalene	Paradichlorobenzene	0.30
55	1906	β-Phenethyl alcohol	Ethanol	1.28
56	2013	Methyleugenol	Terpenoid	3.99
57	2599	15-Hydroxy-α-muurolene	sesquiterpenoids	0.45
Monoterpene and aromatics				4.86
Monoterpenoid				69.64
Sesquiterpene				5.37
Fatty alcohols				3.85
Ketone				6.87
Ester and alkanes				3.12
Others				6.25
Total				99.96
